# Employment of a high throughput functional assay to define the critical factors that influence vaccine induced cross-variant neutralizing antibodies for SARS-CoV-2

**DOI:** 10.1038/s41598-023-49231-w

**Published:** 2023-12-09

**Authors:** Yue Gu, Bhuvaneshwari Shunmuganathan, Xinlei Qian, Rashi Gupta, Rebecca S. W. Tan, Mary Kozma, Kiren Purushotorman, Tanusya M. Murali, Nikki Y. J. Tan, Peter R. Preiser, Julien Lescar, Haziq Nasir, Jyoti Somani, Paul A. Tambyah, SCOPE Cohort Study Group, Siew-Wai Fong, Siti Naqiah Amrun, Yun-Shan Goh, Matthew Zi-Rui Tay, Angeline Rouers, Zi Wei Chang, Nicholas Kim-Wah Yeo, Yi-Hao Chan, Pei Xian Hor, Chiew Yee Loh, Yuling Yang, Anthony Torres Ruesta, Vanessa Neo, Wendy Yehui Chen, Estelle Yi-Wei Goh, Alice Soh-Meoy Ong, Adeline Chiew Yen Chua, Samantha Nguee, Yong Jie Tang, Weiyi Tang, Joel Xu En Wong, Kenneth G. C. Smith, Laurent Renia, Lisa F. P. Ng, David C. Lye, Barnaby E. Young, Paul A. MacAry

**Affiliations:** 1 Antibody Engineering Programme, Life Sciences Institute, National University of Singapore, Singapore, Singapore; 2 Department of Microbiology and Immunology, Yong Loo Lin School of Medicine, National University of Singapore, Singapore, Singapore; 3 NUH-Cambridge Immune Phenotyping Centre, National University of Singapore, Singapore, Singapore; 4 Antimicrobial Resistance Interdisciplinary Research Group (AMR-IRG), Singapore-MIT Alliance in Research and Technology (SMART), Singapore, 138602 Singapore; 5 School of Biological Science (SBS), Nanyang Technological University (NTU), 60 Nanyang Dr, Singapore, 637551 Singapore; 6 NTU Institute of Structural Biology, Nanyang Technological University, 59 Nanyang Drive, Singapore, 636921 Singapore; 7 Division of Infectious Disease, University Medicine Cluster, National University Hospital, Singapore, Singapore; 8 A*STAR Infectious Diseases Labs, Agency for Science, Technology and Research (A*STAR), Singapore, Singapore; 9 Cambridge Institute of Therapeutic Immunology and Infectious Disease, Jeffrey Cheah Biomedical Centre, University of Cambridge, Cambridge, CB2 0AW UK; 10 Department of Medicine, University of Cambridge School of Clinical Medicine, Cambridge, CB2 0QQ UK; 11 Lee Kong Chian School of Medicine, Nanyang Technological University, Singapore, Singapore; 12 National Centre for Infectious Diseases (NCID), Singapore, Singapore; 13 Tan Tock Seng Hospital, Singapore, Singapore; 14 Yong Loo Lin School of Medicine, National University of Singapore, Singapore, Singapore

**Keywords:** Biological techniques, Biotechnology, Immunology, Molecular biology, Diseases, Medical research

## Abstract

The scale and duration of neutralizing antibody responses targeting SARS-CoV-2 viral variants represents a critically important serological parameter that predicts protective immunity for COVID-19. In this study, we describe the development and employment of a new functional assay that measures neutralizing antibodies for SARS-CoV-2 and present longitudinal data illustrating the impact of age, sex and comorbidities on the kinetics and strength of vaccine-induced antibody responses for key variants in an Asian volunteer cohort. We also present an accurate quantitation of serological responses for SARS-CoV-2 that exploits a unique set of in-house, recombinant human monoclonal antibodies targeting the viral Spike and nucleocapsid proteins and demonstrate a reduction in neutralizing antibody titres across all groups 6 months post-vaccination. We also observe a marked reduction in the serological binding activity and neutralizing responses targeting recently newly emerged Omicron variants including XBB 1.5 and highlight a significant increase in cross-protective neutralizing antibody responses following a third dose (boost) of vaccine. These data illustrate how key virological factors such as immune escape mutations combined with host demographic factors such as age and sex of the vaccinated individual influence the strength and duration of cross-protective serological immunity for COVID-19.

## Introduction

The emergence of SARS-CoV-2 Variants of Concern (VOC) and Variants of Interest (VOI) underlies an urgent requirement to define robust measures of protective immunity that can be utilized to triage at-risk populations and effectively target preventative countermeasures^1–5^. The Pfizer/BioNTech mRNA vaccine BNT162b2 is a leading vaccine approved by the US Food and Drugs Agency (FDA). However, there remains a significant concern about the degree of cross-protection afforded against VOC and VOI based upon multiple reports of break-through infections in partially or fully vaccinated individuals^6–8^. Moreover, there remains an urgent need to identify, triage and boost at-risk groups who have responded poorly to vaccination and/or remain susceptible to severe disease. This is now relevant as the WHO (World Health Organization) has declared that COVID-19 is no longer a public health emergency of international concern (PHEIC) but has stressed the importance of long-term management with associated response, management strategies and measures during this transition period.

Amongst the immunological parameters that have been analysed in human populations that predict protection from SARS-CoV-2 mediated disease, the strength and duration of the neutralizing antibody response has emerged as the strongest correlate for protection from symptomatic SARS-CoV-2 infection^9,10^. The emergence of the Delta strain which was first detected in India in September 2020^11^ indicated a possibility of increased virulence in new variants and underpinned the importance of monitoring their emergence. The Omicron variants now represent the key infective strains of SARS-CoV-2 globally-these were first identified in South Africa November 2021^12–14^. Previous studies have estimated a significantly lower level of protection against Delta compared to the ancestral Wuhan-Hu-1 strain upon completion of a two-dose regime of the BNT162b2 vaccine, and neutralizing responses against Beta and Gamma variants are also significantly impaired^1,4,15,16^. Moreover, several reports employing the isolated Omicron virus and/or Omicron Spike-pseudovirus have indicated a significant reduction in neutralizing antibody titres^4,17–23^.

Given the known immune escape potential of SARS-CoV-2 VOC, there remains an urgent need to evaluate cross-protective serological responses engendered by COVID-19 vaccines. Several studies have reported weaker vaccine responses in elderly populations and in males^2,3,16,24–26^. In addition, a small number of published reports have described differential responses when the seropositive population is stratified according to ethnicity, body mass index (BMI), and pre-existing comorbidities such as hypertension^24,27–29^. We describe a comprehensive analysis of the cross-neutralizing and cross-binding serological responses engendered by the BNT162b2 vaccine against key viral antigens from Wuhan-Hu-1, VOC (Alpha, Beta, Gamma, Delta, Omicrons BA.1, BA.2, BA.4/5, XXB and XBB 1.5) and two VOI (Epsilon and Kappa) in an Asian cohort comprised primarily of ethnic Chinese, Malay and Indian volunteers. These data have important implications for our understanding of the key factors that influence vaccine-induced immunity for SA RS-CoV-2 VOC and VOI.

## Results

### Study design

**Figure 1:**
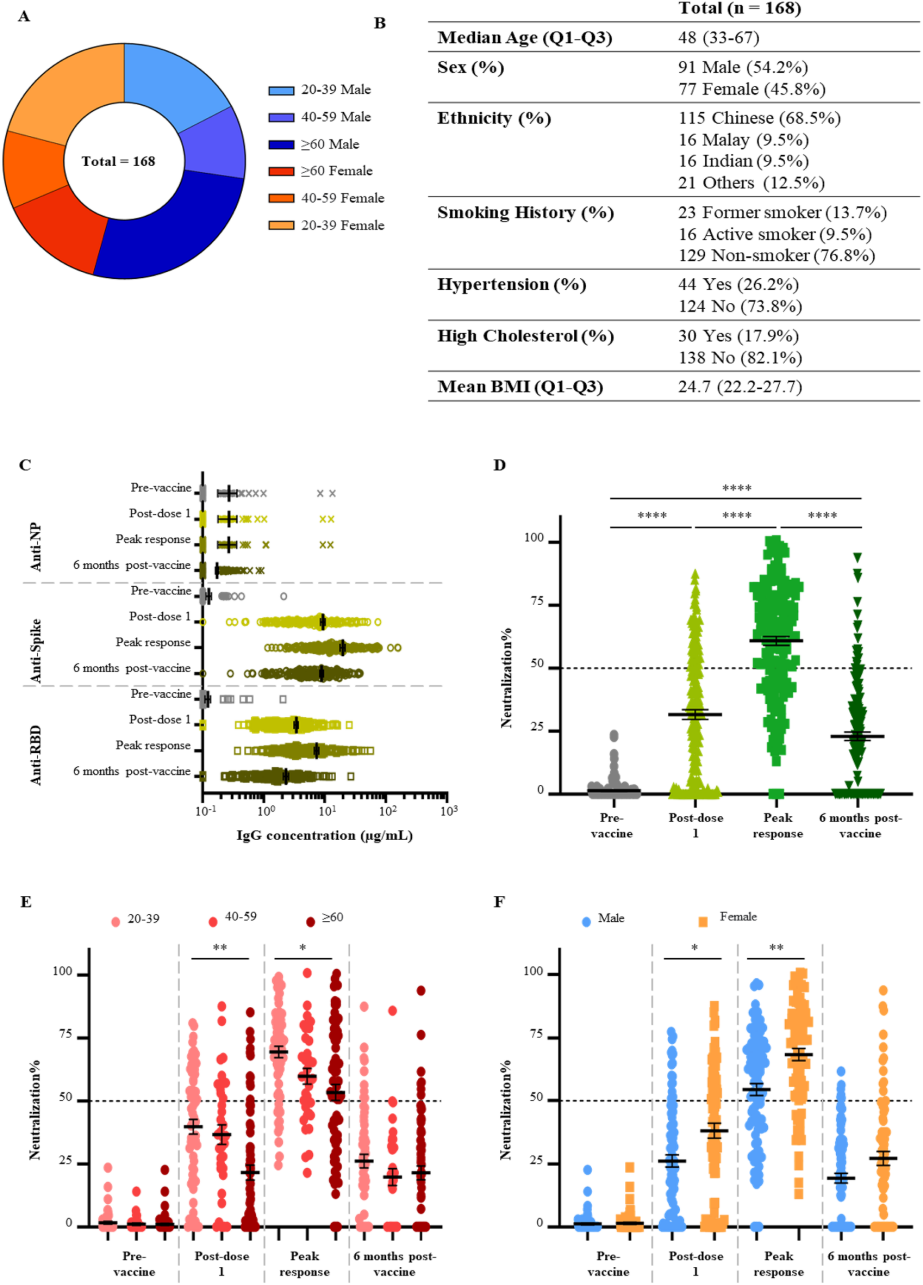
Study cohort and serological response against the Wuhan-Hu-1 strain. (**A**) Demographic distribution of cohort. (**B**) Detailed information on study cohort according to age, gender, ethnicity, and comorbidities (**C**) Quantification of serological IgG antibodies at four timetpoints against SARS-CoV-2 NP, spike and RBD. (**D**) Neutralizing response at four timepoints assessed by PVNT. Multiple linear regression was employed to evaluate the response at each timepoint. The model contained all parameters described in this study. Results were statistically significant for (**E**) age groups and (**F**) sex. All results were analyzed by Kruskal–Wallis test followed by Dunn’s test, N = 168, data represented as mean ± SEM, *p < 0.05, **p < 0.01, ***p < 0.001, ****p < 0.0001.

Longitudinal blood samples taken pre-vaccination, 3 weeks post first dose, 3 months post first dose (peak response), and 6 months post first dose (long-term response) from a total of 168 participants (medium age 48, 45.8% female) were analyzed (Fig. 1A,B). All participants were given at least two doses of the Pfizer/BioNTech BNT162b2 vaccine at 21-day intervals. In this cohort, a subset of 25 individuals had received a third dose of same vaccine (i.e., booster dose), and plasma were taken 1 to 3 months after this dose. None of the participants were diagnosed with COVID-19 previously.

### Serological IgG binding to SARS-CoV-2 antigens

SARS-Cov2 antigens and receptor protein (ACE2-Fc), were expressed, purified (Supplementary Fig. 1). Plasma IgG antibodies targeting SARS-CoV-2 full spike (N-terminal domain (NTD) + S1 subunit + S2 subunit), Spike-receptor binding domain (RBD) and Nucleocapsid protein (Fig. 1C) were measured using an Enzyme-Linked Immunosorbent Assay (ELISA) employing fully human or humanized antibodies (for Spike, RBD and Nucleocapsid) to construct comparative standard curves and enable accurate quantitation (Supplementary Fig. 2). Anti-Nucleocapsid IgG remains negative throughout the study period for most participants (Fig. 1C). Both individuals who exhibited high pre-vaccination anti-Nucleocapsid IgG were negative for anti-Spike and anti-RBD IgG (Supplementary Fig. 3), indicating a high chance of cross-reactivity from previous infections by other coronaviruses. Both anti-Spike IgG and anti-RBD IgG increased significantly after two doses of the vaccine, with mean concentrations of 19.7 µg/mL and 7.4 µg/mL respectively at 2 months post second dose (Fig. 1C). We observed a significant decrease in binding titers of IgG antibodies analyzed 6 months post-vaccination, but these titers were still higher than those measured pre-vaccination (Fig. 1C).

### Serological IgG neutralizing SARS-CoV-2 spike-pseudovirus

A SARS-CoV-2 spike pseudovirus neutralization test (PVNT) was employed to measure neutralizing activity at all time points. A significant neutralizing response was elicited by the first dose of BNT162b2 (mean 31.6% neutralization) and it improved further after the second dose with a mean of 60.9% neutralization (Fig. 1D). However, the neutralizing response reduced significantly to an average of 22.9% at 6 months post-vaccination. Vaccinated individuals below 40 years old responded strongly after a single vaccine dose compared to those aged 60 years and above. This observed difference was reduced after a second dose of vaccine. At 6 months post-vaccination, there was a marked decrease in the neutralizing antibody response in all age groups tested (Fig. 1E). In addition, we observed that females made stronger neutralizing responses after either one or two doses of vaccine (Fig. 1F), as previously reported.

### Development and calibration of an ELISA assay to measure neutralizing antibody responses against viral variants

**Figure 2:**
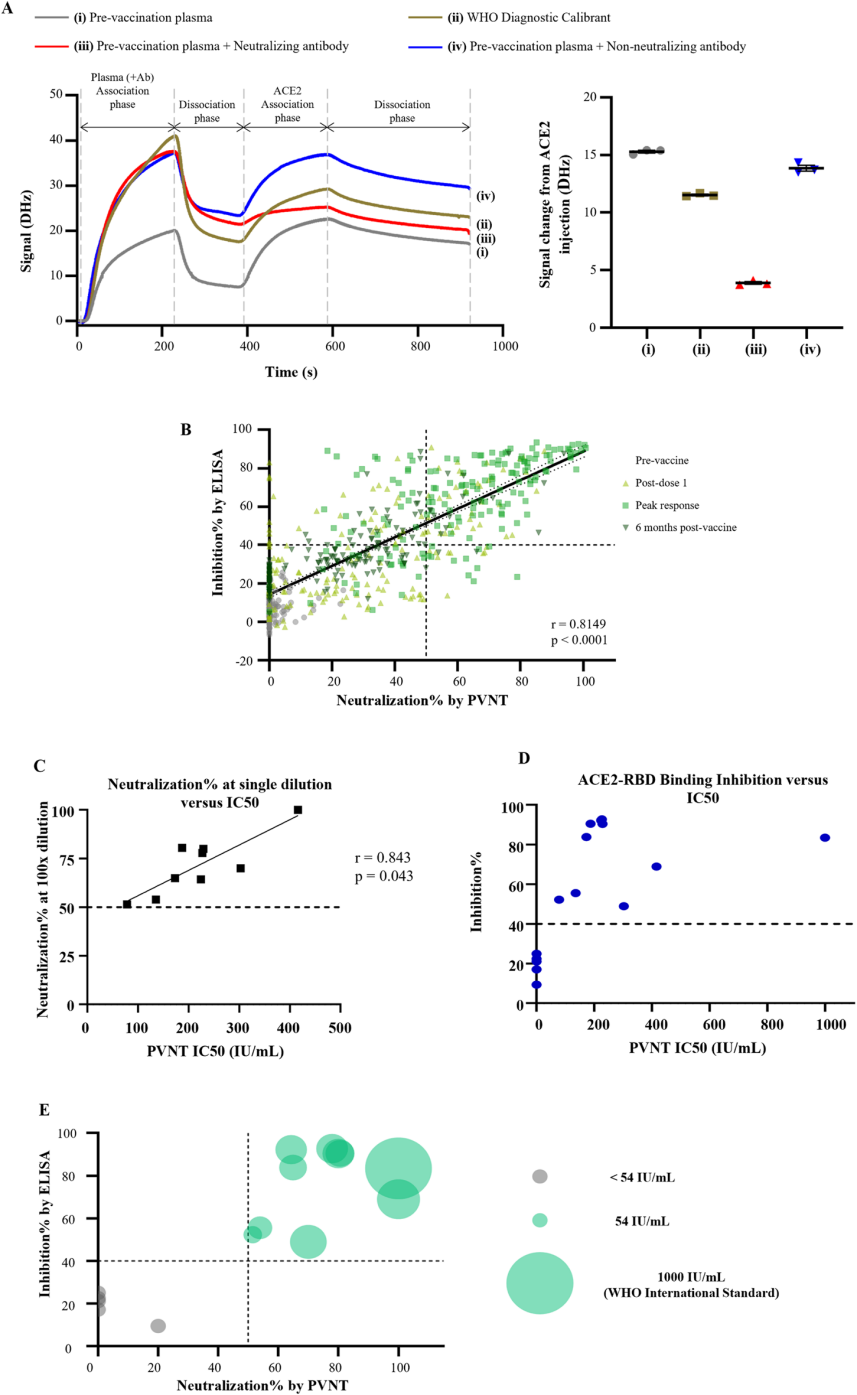
Measurement of ACE2-RBD inhibitory response in a surrogate neutralization assay. (**A**) Left panel: the effect of (i) pre-vaccination plasma, (ii) WHO diagnostic calibrant, (iii) a SARS-CoV-2 neutralizing antibody, and (iv) a SARS-CoV-2 non-neutralizing antibody on inhibiting the interaction between ACE2 and RBD were examined using quartz crystal microbalance technology. Right panel: After RBD interaction with plasma (and antibodies), subsequent signal change during the ACE2 association phase were calculated as a parameter negatively correlated with ACE2 inhibition efficiency. (**B**) Correlation between neutralizing response by PVNT and ACE2-RBD binding inhibition for Wuhan-Hu-1 RBD at four timepoints were modelled using simple linear regression. N = 672. Pearson’s correlation coefficients and p-values are shown. (**C**) Pearson’s coefficients and p-values were calculated for the correlation between the IC50 values and neutralization% at 100× dilution of sample (n = 9). (**D**) ACE2-RBD binding inhibition response was plotted against the PVNT-derived IC50 values after calibration with WHO international standards (n = 13). IC50 values are not available (below 50 IU/mL) for all samples below the arbitrary threshold of 40% ACE2-RBD binding inhibition (n = 4). (**E**) ACE2-RBD binding inhibition response against Wuhan-Hu-1 RBD was plotted against the neutralizing response measured by PVNT for 15 samples. Size of point represents concentration in IU/mL after calibration with WHO international standards.

To augment our analyses of neutralizing antibodies based upon PVNT, we tested the ability of antibodies in volunteer plasma samples to block the interaction between ACE2 and Spike-RBD. Using QCM to map the kinetics of antibody-Spike-RBD binding plus ACE2 association/dissociation, we observed the real-time inhibition of these interactions by neutralizing antibodies (Fig. 2A). Specifically, when pre-vaccination plasma or a non-neutralizing monoclonal human antibody with defined binding activity for Spike-RBD^30^ is employed and allowed to interact with the immobilised Spike-RBD, injected ACE2 was not blocked/occluded and thus able to interact with the Spike-RBD ligand, as indicated by the signal change of 15.3 Hz or 13.87 Hz respectively over time. In contrast, the impact of neutralizing antibodies is shown through the reduction in signal change when ACE2 is injected after the addition of plasma containing a defined neutralizing human antibody specific for Spike-RBD^31^ (11.54 Hz) analyzed over the same time period. This observed inhibitory effect is increased with a WHO Diagnostic Calibrant (pooled convalescent plasma) (3.89 Hz), indicating a strong inhibition of ACE2 Spike-RBD interactions (Fig. 2A). Based upon these observations, we optimized an ACE2-RBD binding inhibition assay. This ELISA-based surrogate viral neutralisation test measures antibodies that block ACE2 Spike-RBD interactions and correlates strongly with neutralizing responses measured by PVNT for the wildtype strain (Fig. 2B,C). The ACE2 inhibition ELISA has a sensitivity of 87.8% and specificity of 81.3% at 40% inhibition when compared with the established 50% cut-off for PVNT that is utilised to defined strong neutralizers. This threshold is employed hereafter to define the neutralizing response in our volunteer cohorts^32^ (Fig. 2C). Moreover, we show that the observed neutralizing titres measured by this ELISA can be converted into international units (IU) through the employment of WHO reference materials and defined high, moderate, and low neutralizing samples (IC_50_ measured by PVNT) (Fig. 2D). It has been estimated that 50% protective neutralization level is approximately equivalent to 54 IU/mL^9^. In this study, all samples below 54 IU/mL exhibited less than a 40% inhibition of ACE2-RBD binding (Fig. 2E). In addition to showing a strong correlation for the wildtype strain, we show that this assay can be multiplexed by incorporating the multiple SARS-CoV2 viral variants. The ACE2-RBD binding-inhibition percentages for 4 of the major VOCs; Wu-H1, Delta, Omicrons BA.1 and BA.2 were compared to that of the neutralising antibody responses obtained from PVNT. The comparison showed significantly strong correlations for all 4 VOCs that were evaluated (Supplementary Fig. 7).

### ACE-2 RBD binding kinetics and serological response to SARS-CoV-2 variants

**Figure 3:**
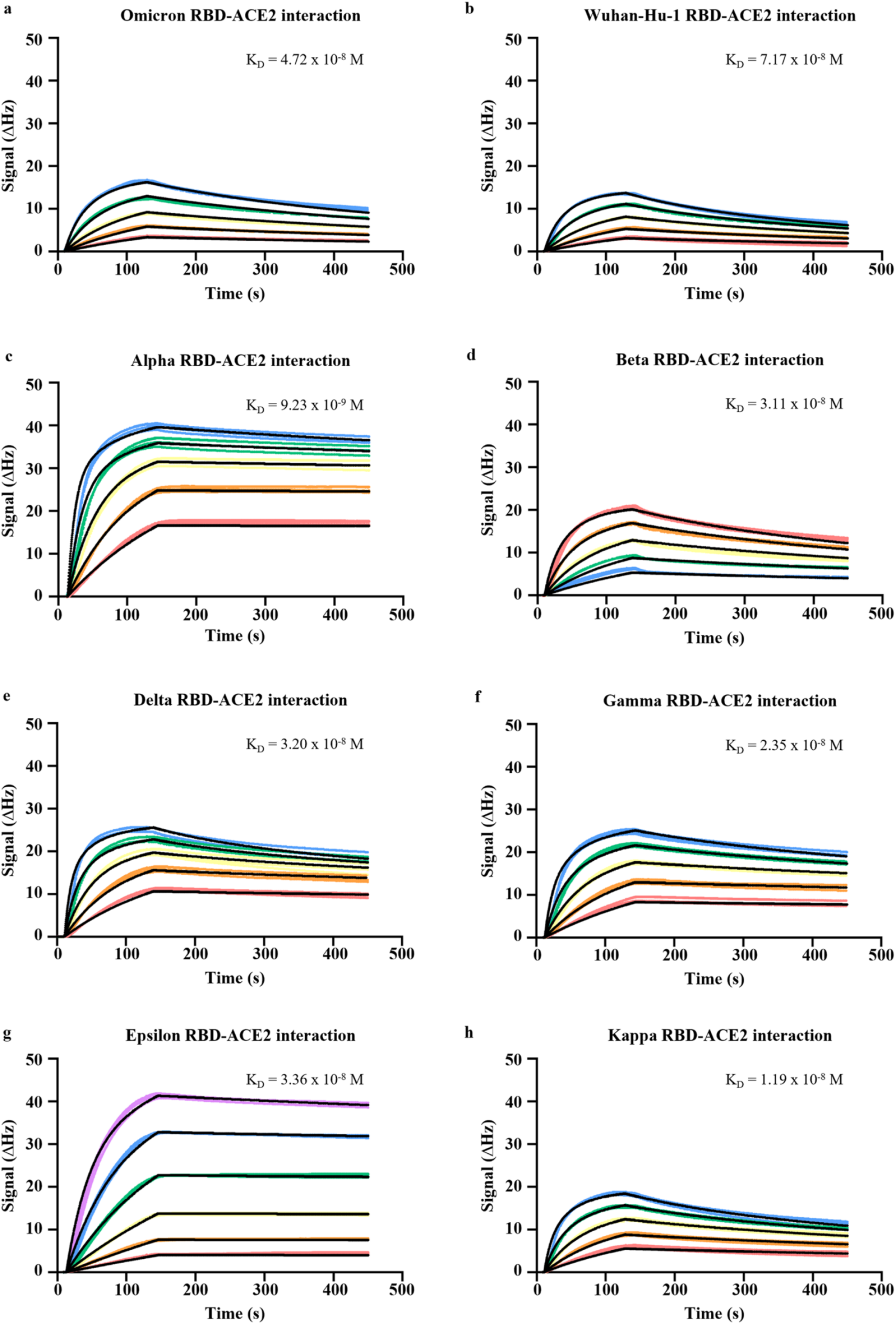
Interaction kinetics between RBD and ACE2. Binding kinetics between ACE2 and (**a**) Omicron RBD, (**b**) Wuhan-Hu-1 RBD, (**c**) Alpha RBD, (**d**) Beta RBD, (**e**) Delta RBD, (**f**) Gamma RBD, (**g**) Epsilon RBD, or (**h**) Kappa RBD immobilised on chips were studied using studied using quartz crystal microbalance technology. Dissociation equilibrium constants (K_D_) was estimated based on three technical repeats.

**Figure 4:**
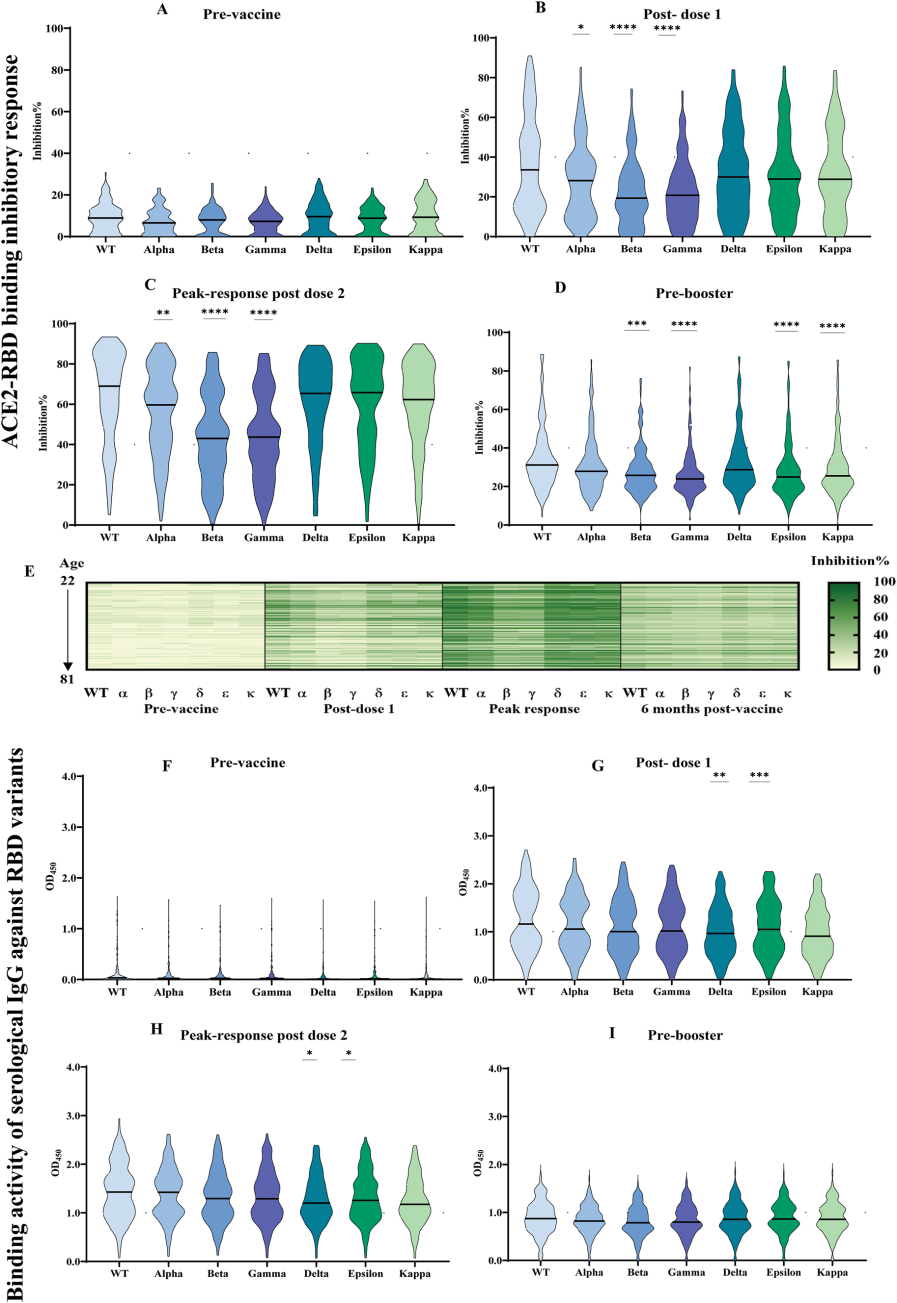
ACE2-RBD inhibitory response and binding activity of serological IgG against RBD variants. The ACE2-RBD binding inhibitory response for seven RBD variants at (**A**) pre-vaccination, (**B**) post–dose 1, (**C**) peak response post-dose 2, and (**D**) 6 months post-dose 1 were evaluated by ELISA. (**E**) Response at four timepoint were represented as a heatmap. Data analyzed by Kruskal–Wallis test followed by Dunn’s test. Data represented as mean ± SEM, *p < 0.05, **p < 0.01, ***p < 0.01, ****p < 0.0001. Serological IgG binding activity to seven RBD variants (**F**–**I**) at four timepoints were measured by ELISA. Results were analyzed by Kruskal–Wallis test followed by Dunn’s test. N = 168, *p < 0.05, **p < 0.01, ***p < 0.001.

Several studies have reported that RBD variants display variable binding affinities to human ACE2 and this has been linked to the different polymorphic regions. Notably, when comparing the different RBD variants, the Omicron RBDs which harbour the largest number of mutations, show the strongest affinities compared to the wild type or D416G mutation RBD. These studies have also suggested that differences in RBD-ACE2 interactions are linked to differing host susceptibility and infection^33^. To further strengthen the serological profiling of the donor plasma samples, QCM was employed to study the interaction kinetics between ACE2 and other RBD variants (Alpha, Beta, Gamma, Delta, Epsilon and Kappa and Omicron), in addition to the Wu-H1 strain (Fig. 3a–h). All equilibrium dissociation constants (K_D_) were measured in the 10^–8^ to 10^–9^ M range (Fig. 3a–h). Following the kinetics analysis, ACE2-RBD inhibition activity against these RBD variants were evaluated using the developed ELISA-based assay. Overall, the ACE2-RBD inhibition activity of vaccinee plasma to the Wuhan-Hu-1 strain (labelled as wild type, WT) is predictably one of the strongest amongst all variants tested. We observed significant and consistently lower levels of ACE2-RBD inhibition activity against the Beta and Gamma strains compared to the Wuhan-Hu-1 strain at all timepoints tested until 6 months post-vaccination (Fig. 4A–E). In contrast, this trend is not observed in the binding activity to variant Spike-RBDs (Fig. 4F–I). At peak response after the second dose, the mean inhibition against all variants scored above 40%, indicating a significant degree of cross-neutralization/protection. Concordant with our observations with PVNT, the percentile inhibition of RBD-ACE2 binding for all variants also decreased at 6 months post-vaccination.

Females also showed a better response against all variants compared with males, in agreement with our PVNT data (Supplementary Tables 1, 2). Compared to those below 40 years old, vaccine recipients aged 60 years and above exhibited a significantly lower level of antibodies that block ACE2 interactions with the Delta variant of Spike-RBD at peak response (Supplementary Table 2). We also observed a small but significantly stronger peak response against the Gamma variant in our Indian vaccinees compared to those of Chinese ethnicity. In addition, borderline differences against the Alpha and Beta variants were detected between these two cohorts. While needing validation in larger cohorts, this raises the possibility that significant ethnic differences in vaccine responsiveness may exist. Compared to non-smokers, a higher percentile inhibition of ACE2-RBD binding was observed in former but not active smokers (Supplementary Table 2). BMI and comorbidities such as hypertension and high cholesterol had no impact on the observed neutralizing titres against the VOC and VOI tested at all time points (Supplementary Tables 1–3).

### Serological response to key SARS-CoV-2 variants including five recent Omicron variants

**Figure 5:**
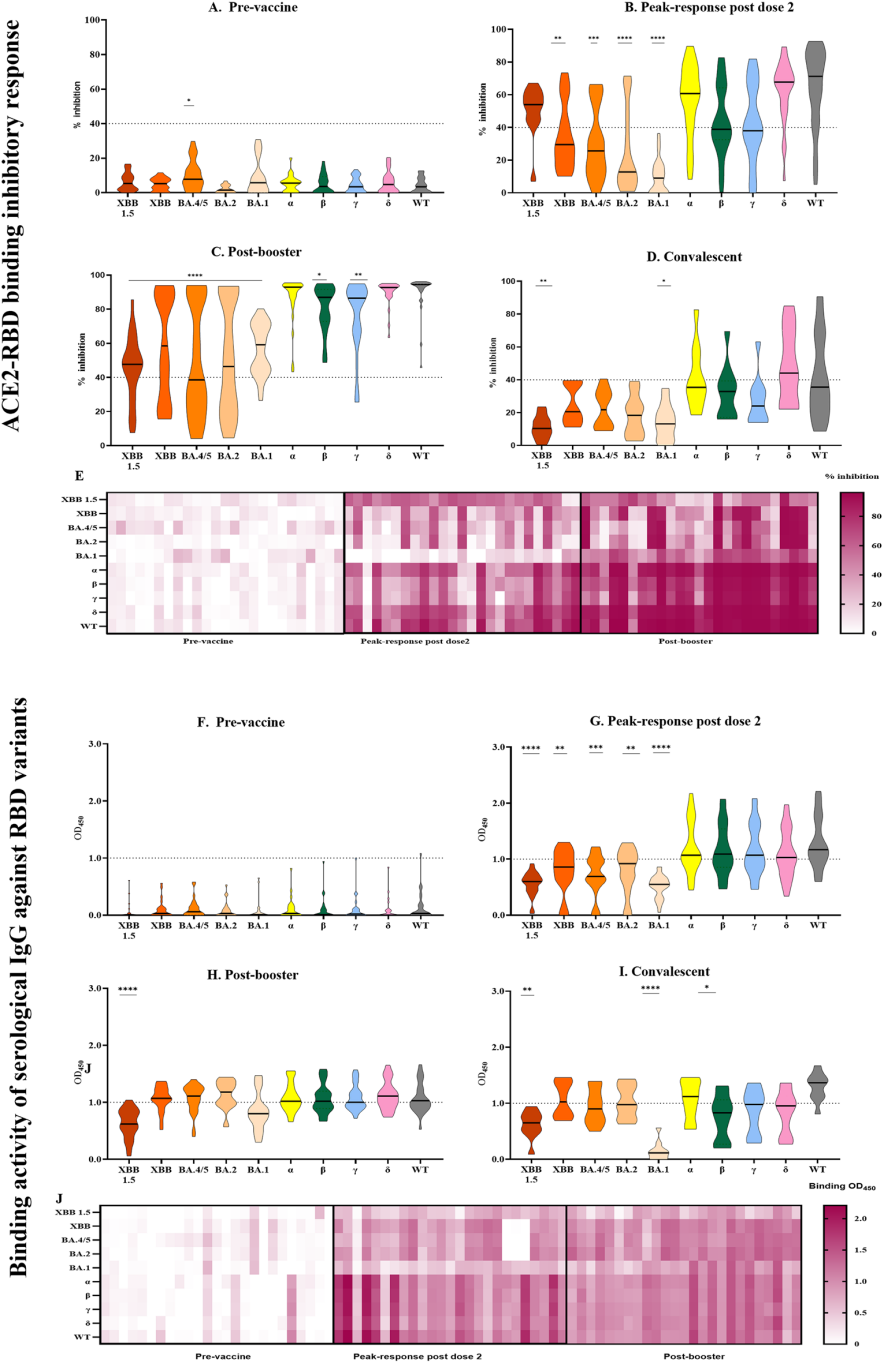
ACE2-RBD inhibitory response and binding activity against key VOCs including 5 Omicron variants in vaccinees and convalescent individuals. ACE2-RBD binding inhibitory response to Wuhan-Hu-1 (WT) RBD and all VOC RBDs at (**A**) pre-vaccination, (**B**) peak-response post dose 2, (**C**) post-booster dose, and (**D**) post recovery from COVID-19 were measured by ELISA. (**E**–**H**) Serological IgG binding activity were also evaluated at these timepoints. (**I**) The ACE2-RBD inhibitory response and (**J**) RBD-binding activity at three timepoints were summarized for vaccinees. Response at each timepoint were analyzed by Kruskal–Wallis test followed by Dunn’s test. N = 25 for (**A**–**C**,**E**–**G**,**I**,**J**). N = 10 for (**D**,**H**). Data represented as mean ± SEM, *p < 0.05, **p < 0.01, ***p < 0.01, **** p < 0.0001.

The emergence of recent omicron variants with higher transmissibility and immune escape potential, led to a key question regarding strength and durability of immune response elicited by the vaccine against these omicron variants. To address this knowledge gap in protective immunity especially in vulnerable populations, the serological responses against the key VOC including 5 of the recent Omicron variants (BA.1, BA.2, BA.4/5, XBB and XBB 1.5) were evaluated in a group of 10 recovered convalescent individuals, and a subset of 25 vaccinees with age > 60 at three timepoints: pre-vaccination, peak response after second dose of vaccine, and 1 to 2 months after the booster dose (Fig. 5). The IgG binding activity to the Omicron RBDs were reported as follows: 68.0% and 84.0% of vaccinated plasma samples detected IgG binding activity to XBB 1.5, XBB and BA.4/5 respectively after the second dose of vaccine (Fig. 5B), and 76.0% and 68.0% to BA.2 and BA.1 respectively (Fig. 5B), this was significantly lower compared to the Wuhan-Hu-1 strain and all other VOC, except in comparison with the Beta variant in convalescent individuals. This segregation in response by variants was less prominent after the booster dose (Fig. 5C).

In concordance with the observed reduction in binding activity, ACE2-RBD inhibition activity of post-dose 2 and convalescent plasma samples against all 5 Omicron RBDs was significantly lower than the ancestral strain and all other VOC (Fig. 5G,I). Before the emergence of the Omicron variants, immune escape effects were often reported to be the most potent in Beta and Gamma variants^4,34^. Here, we show that the post-dose 2 ACE2-RBD inhibition responses to the 5 Omicron variants are inferior to both Beta and Gamma variants (p < 0.001), though to a lesser extent compared to Wuhan-Hu-1, Alpha, and Delta variants (p < 0.0001). A similar trend was observed in the convalescent group (Fig. 5I). Again, we highlight that there is a significant increase in the ACE2-RBD inhibition responses against the Omicron variants. This is relatively comparable to the other variants after the booster dose of vaccine (Fig. 5H).

Based on the comparison between vaccinees at post-dose 2 peak response and convalescent individuals, we observed that the neutralizing responses against the Wildtype, Gamma, Alpha, and XXB 1.5 variants in the convalescent group were significantly lower than in the vaccinated group. In contrast for total IgG binding, only the Beta and BA.1 variants showed significant differences between the convalescent and vaccinated groups. This may be expected as many of the PCR-positive individuals included in this study only presented mild symptoms An additional vaccine boost led to a marked increase in the IgG binding response and neutralizing responses against all variants including Omicron (Fig. 5H,J).

## Discussion

The successful rollout of national vaccination programmes has been crucial in reducing mortality from pandemic COVID-19 and will continue to have an important impact once SARS-CoV-2 has become endemic. To date, 60.5% have received at least one dose of vaccine globally^35^. The long-term effectiveness of this strategy is dependent on the degree of cross-protection afforded for VOC, and the Pfizer/BioNTech BNT162b2 represents one of the key platforms employed.

In this Asian cohort, we observed a good neutralizing response for Wuhan-Hu-1 after both doses of BNT162b2, with a mean 60.9% neutralization by PVNT, translating into an average IC50 of 138.6 IU/Ml. However, at peak response 30.3% (51 out of 168) of the vaccinees exhibited a neutralizing response below 50%. In addition, the mean neutralizing titres across all groups fell below 23% at 6 months post-vaccination, indicating a need for an additional booster dose. Our Wuhan-Hu-1 RBD-ACE2 binding inhibition ELISA result also illustrates the effectiveness of the booster dose. Among the subset of vaccinees whose post-booster plasma samples were analyzed, 16.0% (4 out of 25) showed sub-optimal ACE2 inhibition (below 40% inhibition) at peak response after the second dose. All these individuals showed an enhancement in ACE2 inhibitory response (to over 40% inhibition) after the booster dose.

In concordance with other studies, we report a significantly weaker ACE2-RBD inhibition response in the group aged 60 years and above for the Delta variant, and this qualitative readout was not matched by quantitative differences in IgG binding to SARS-CoV-2 antigens. These findings are also consistent with reports of vaccine break-through infections with the Delta variant^7,8^. In addition, we observed stronger neutralizing responses among female vaccine recipients versus to males across all viral variants tested, as previously reported in Europeans^16,24,25^. We highlight that all vaccinees exhibited a significant reduction in their ACE2-RBD inhibition response at 6 months post-vaccination regardless of age group (p = 0.5504), sex (p = 0.1103), or any other categories analyzed.

Long lived PCs and serum IgG correlated with protection, and the decline in antigen-specific IgG over time may be a concern. This reflects, however, only one of several of key components contributing to effective immunological memory. Memory B cells also provide long-term memory that is qualitatively different to that reflected in serum IgG titres. Memory B cell selection results in their recognition of a relatively broad range of antigenic specificities, in contrast to long-lived PCs that are more stringently selected for affinity and specificity^36,37^. Thus, memory B cells are likely to provide a response better able to counter new VOC such as Omicron, in a manner not reflected in serological assays. In addition to this, T cell memory also plays an important role in protection. The way in which these aspects of memory are impacted by different vaccine schedules, and by infection itself^38^, in different ethnic groups, will be important in optimising vaccine strategies.

We extended our study to compare responses to the early SARS-CoV-2 variants with the recent Omicron variants, including the highly transmissible recent XBB 1.5. Serological responses to the Omicron variants were markedly reduced compared to those to the ancestral Wuhan-Hu-1 strain and other VOC, consistent with reports from other countries^17–23^. This illustrates that the immune escape effect of Omicron is greater than that of the Beta and Gamma variants. While the increased protection against Omicron from booster doses of vaccination is encouraging, follow-up studies to determine the duration of this booster effect in specific ethnic groups are required.

Having compared the ACE2-RBD inhibition responses across ethnicities, we observed a small difference in a single timepoint. But overall, this difference is minor compared to age and sex, suggesting that ethnicity is not a major factor in determining the strength and durability of antibody response to the vaccine. Given the observed reduction in neutralizing antibody titres in all groups at 6 months post-vaccination, plus the overall augmentation in cross-protective response to all VOC including this argues strongly for the implementation of vaccine booster shots that augment the degree of cross-protective responses particularly in at-risk groups.

## Conclusion

This study highlights the need for vigilance in monitoring the emergence of VOC, and of assessing the efficacy of vaccination programmes in protecting against them, particularly where individuals who are above 60 years old and male are less well-protected by neutralizing antibodies.

## Materials and methods

### Study design, ethical statement, and sample collection

The vaccinated participants were recruited under the COVID-19 PROTECT study (2012/00917). The convalescent plasma samples were collected from subjects who were diagnosed with COVID-19 by positive polymerase chain reaction (PCR) results (study 2020/00120). All participants provided written informed consent in accordance with the Declaration of Helsinki for Human Research. Ethics committee of National Healthcare Group (NHG) Domain Specific Review Board (DSRB) Singapore gave ethical approval for this work.

All vaccinated participants received two doses of the Pfizer/BioNTech BNT162b2 mRNA vaccine at 21 days apart. Four plasma samples were collected from each participant: on the day of first dose, before vaccination (i.e., pre-vaccination); on the day of second dose, before vaccination (i.e., post-dose one); 3 months after the first dose (i.e., peak response); and 6 months after the first dose. In addition, plasma sample from a fifth timepoint at 1 to 3 months after the booster dose (i.e., third dose) were collected from 27 individuals. To analyze the response to vaccination in the general population, participants with known SARS-CoV-2 infection history and participants under immunosuppressive treatments were excluded. A total of 699 plasma samples from 168 participants were included in this study.

Ten convalescent plasma samples were collected 1 to 3 months after diagnosis. All convalescent volunteers had recovered prior to sample collection.

### Expression and purification of SARS-CoV-2 antigens and receptors

SARS-CoV-2 Spike hexapro, RBD and ACE2 were purified as described elsewhere^39^. RBD variants were made using RBD as the template and KLD enzyme mix (NEB) and expressed and purified using the same method as for RBD. Primers used are listed in Supplementary Table 5.

Gene encoding SARS-CoV-2 Nucleocapsid (Biobasic) was cloned into Pnic28, expressed in BL21 (DE3), and purified from soluble fraction using cOmplete™ his tag purification resin (Roche).

### Quantitative ELISA/RBD variant binding ELISA

Antigens were diluted in 1× PBS and coated onto 96-well flat-bottom maxi-binding immunoplates (SPL Life Sciences #32296) by incubating at 4 °C overnight. Plate was washed three times with washing buffer (1× PBS with 0.05% Tween-20) and blocked with blocking buffer (3% bovine serum albumin in washing buffer) for 60 min incubation. Plasma samples were diluted 200-times and 5000-times in blocking buffer and added to the plates. To estimate the concentrations of anti-spike and anti-RBD antibodies, an RBD-specific human monoclonal IgG antibody named LSI-COVA-015 isolated from COVID-19 convalescent patient was diluted to a series of concentrations ranging from1 ng/mL to 1 µg/mL and added. Similarly, a nucleocapsid-specific monoclonal IgG antibody named LSI-COVANC-D generated from hybridoma cloning^40,41^ was added at the same concentrations to estimate the concentration of anti-nucleocapsid antibodies in the plasma samples. After 60 min incubation, plate was washed and incubated with goat anti-human IgG-HRP antibodies (Invitrogen #31413, diluted 10,000-times in blocking buffer) for 50 min, protected from light. Plate wash step was repeated and TMB substrate (Thermo Scientific #34029) was added. After 3 min incubation, reaction was stopped with 1 M H_2_SO_4_ and optical density at 450 nm (OD_450_) were recorded. Standard curves were constructed using the reference antibodies from 100 to 1 ng/mL, and the concentration of antigen-specific IgG antibodies in plasma samples were calculated via interpolation.

For the RBD variant binding ELISA, plasma samples were tested at 100-times dilution. A negative control (100-times diluted heat-inactivated FBS) and a positive control (ACE2-Fc at 5 µg/mL in negative control) were included for each variant in each plate.

### SARS-CoV-2 pseudotyped lentivirus production

Reverse transfection methodology was employed to generate pseudotyped viral particles expressing SARS-CoV-2 Spike proteins, using a third-generation lentivirus system. A total of 36 × 10^6^ HEK293T cells were transfected with 27 µg pMDLg/Prre (Addgene, #12251), 13.5 µg Prsv-Rev (Addgene, #12253), 27 µg Ptt5LnX-WHCoV-St19 (SARS-CoV-2 Spike) and 54 µg Phiv-Luc-ZsGreen (Addgene, #39196) using Lipofectamine 3000 transfection reagent (Invitrogen, #L3000-150) and cultured in a 37 °C, 5% CO_2_ incubator for three days. At day 4, the viral supernatant was collected and filtered through a 0.45 µm filter unit (Merck). The filtered pseudovirus supernatant was concentrated using 40% PEG 6000 via centrifugation at 1600*g* for 60 min at 4 °C. Lenti-X p24 rapid titer kit (Takara Bio, #632200) was used to quantify the viral titers, as per manufacturer’s protocol.

### Pseudovirus neutralization test (PVNT)

ACE2 stably expressed CHO cells were seeded at a density of 5 × 10^4^ cells in 100 µL of complete medium [DMEM/high glucose with sodium pyruvate (Gibco, #10569010), supplemented with 10% FBS (Hyclone, #SV301160.03), 10% MEM Non-essential amino acids (Gibco, #1110050), 10% geneticin (Gibco, #10131035) and 10% pencillin/streptomycin (Gibco, #15400054)], in 96-well white flat-clear bottom plates (Corning, #353377). The cells were cultured in 37 °C with humidified atmosphere at 5% CO_2_ for 24 h. The next day, subject plasma samples were diluted to a final dilution factor of 80 with sterile 1× PBS. The diluted samples were then incubated with an equal volume of pseudovirus at a concentration of 2 × 10^6^ IFU/mL to achieve a total volume of 50 µL, at 37 °C for 1 h. The pseudovirus-plasma mixture was added to the CHO-ACE2 monolayer cells and left incubated for 1 h to allow pseudotyped viral infection. Subsequently, 150 µL of complete medium was added to each well for a further incubation of 48 h. The cells were washed twice with sterile PBS. 100 µL of ONE-glo™ EX luciferase assay reagent (Promega, #E8130) was added to each well and the luminescence values were read on the Tecan Spark 100 M. The percentage neutralization was calculated as follows:

$$Neutralization\mathrm{\% }=\frac{Readout \; \left(unknow\right)-Readout \; \left(infected \; control\right)}{Readout \; \left(uninfected \; contorl\right)-Readout (infected \; control)}\times 100\%$$

### PVNT-derived IC50 values (IU/Ml) using calibrated anti-SARS-CoV-2 Immunoglobulin WHO international standard

The ID50 (Inhibitory dilution factor at 50% neutralization) values obtained from the PVNT were converted to IC50 (inhibitory concentration at 50% neutralization) values, using the WHO international standard for anti-SARS-CoV-2 immunoglobulin (20/136). The PVNT assay was conducted as described above. The pseudovirus mixture was incubated with eight serial fivefold dilutions of international standard (1:100 start dilution). The values were then plotted and ID50 was determined. As per WHO’s protocol, 20/136 was assigned an arbitrary value of 250 IU/ampoule (1000 IU/mL) for neutralizing activity. A calibration factor was derived based on ID50 converted to IU/mL (1000/ ID50). Following this, ID50 values were similarly obtained from nine vaccinee samples and three pooled plasma samples from the WHO reference panel (20/150, 20/148, 20/140) incubated with three serial fivefold dilutions (1:100 start dilution). The ID50 values were then converted to IC50 (IU/mL) by multiplying the calibration factor.

### RBD variant binding ELISA

The binding ability of plasma samples to RBD variants were tested using an ELISA protocol similar to the one described earlier. RBD variants were coated at 1 µg/mL and plasma samples were tested at 100-times dilution. A negative control (100-times diluted heat-inactivated FBS) and a positive control (ACE2-Fc at 5 µg/mL in negative control) were included for each variant in each plate. Reported OD_450_ was calculated by subtracting the background OD_450_ of diluted plasma binding to blocking buffer from OD_450_ of diluted plasma binding to RBD variants.

### ACE2-RBD binding inhibition ELISA

The ability of plasma samples to inhibit the binding interaction between ACE2 and RBD variants were evaluated using a protocol similar to the RBD variant binding ELISA. Wuhan-Hu-1, Alpha, Beta, Gamma, and Delta RBD were coated at 1 µg/mL. Wuhan-Hu-1 RBD and Omicron RBD were coated at 2 µg/mL. Wuhan-Hu-1 RBD were coated at two concentrations for calibration of Omicron RBD results to account for the difference in coating concentrations. Plasma samples were tested at 5-times dilution, and the secondary antibody used was ACE2-Peroxidase (conjugated using Peroxidase-labelling kit-NH_2_, Abnova #KA0014), at 600 ng/mL for Omicron, or 300 ng/mL for all other RBD variants. A negative control (5-times diluted heat-inactivated FBS) and a positive control (ACE2-Fc at 100 µg/mL in negative control) were included for each variant in each plate. Inhibition% was calculated using the following formula:

$$Inhibition\mathrm{\% }=\frac{Readout \; \left(negative\; control\right)-Readout \;\left(sample\right)}{Readout\; \left(negative \;control\right)}\times 100\%$$

### Interaction kinetics

Binding kinetics between human ACE2 receptor and SARS-CoV-2 RBD were measured using the Attana Cell 200 (Attana AB), which employs quartz crystal microbalance technology. Standard amine coupling chemistry was used to immobilise Wuhan-Hu-1 RBD or Omicron RBD to LNB-carboxyl sensor chips. Chips were stabilised at a flow rate of 20 µL/min at 22 °C using HBST as running buffer. Triplicate injections were made with human ACE2-Fc at five concentrations followed by regeneration with 10 Mm Glycine (Ph 2).

To examine inhibition of this interaction by SARS-CoV-2 neutralizing antibodies, a second experiment was performed using a fresh LNB-carboxyl chip immobilised as previously with Wuhan-Hu-1 RBD. Triplicate injections were made, in randomized order, over the stabilised surface using 20-times diluted pre-vaccination human plasma, either alone or spiked with 1.67 × 10^–8^ M neutralizing antibody COVA2-39^31^; 1.67 × 10^–8^ M non-neutralizing antibody, LSI-COVA-15; or 20-times diluted WHO Diagnostic Calibrant (pooled convalescent plasma). After each injection 2.6 × 10^–7^ M ACE2 was subsequently injected over the surface and allowed to dissociate before regeneration, as described previously.

Negligible noise (< 3 Hz) was detected on the reference channel. Curve fitting and data analysis was performed using TraceDrawer software.

### Statistical analysis

Continuous demographic data were presented as median with interquartile range (IQR) while categorical demographic data and medical history information were presented as absolute number with proportion (%). Multiple linear regression was used to evaluate the effect of demographic information (age, sex, ethnicity, BMI) and medical history (smoking status, hypertension, high cholesterol) on SARS-CoV-2 antigen-specific IgG concentrations, and neutralizing antibody levels respectively. Neutralizing antibody levels and antigen-specific IgG concentrations across timepoints were evaluated using the non-parametric Kruskal–Wallis test followed by the Dunn’s test to correct for multiple comparisons. Association between the neutralization% from PVNT and inhibition% from ACE2-RBD binding inhibition ELISA was modelled using simple linear regression, and the Pearson’s correlation coefficient was also reported. Binding data and ACE2 inhibition data on RBD variants were compared to the original Wuhan-Hu-1 strains using the Kruskal–Wallis test followed by the Dunn’s test. Results on Wuhan-Hu-1 strain and other RBD variants were compared to the Omicron RBD using the Kruskal–Wallis test followed by the Dunn’s test. All statistical tests were two-tailed when applicable. All experiments were performed with three technical repeats. Mean and standard error of the mean (SEM) are shown in all graphs unless otherwise stated. All analyses were performed using Graphpad Prism 9.0 and can be found in Supplementary Data.

### Supplementary Information

Supplementary Tables.

Supplementary Information 2.

Supplementary Information 3.

Supplementary Information 4.

Supplementary Information 5.

## Data Availability

The datasets generated during and/or analysed during the current study are available from the corresponding author on reasonable request.
