# Supplementary material for: Employment of a high throughput functional assay to define the critical factors that influence vaccine induced cross-variant neutralizing antibodies for SARS-CoV-2
Source: Sci Rep. 2023 Dec 9;13:21810. doi: 10.1038/s41598-023-49231-w (PMC10710454; doi:10.1038/s41598-023-49231-w)
Supplement: Supplementary file 2 — Supplementary Information 2. [file 41598_2023_49231_MOESM2_ESM.docx]

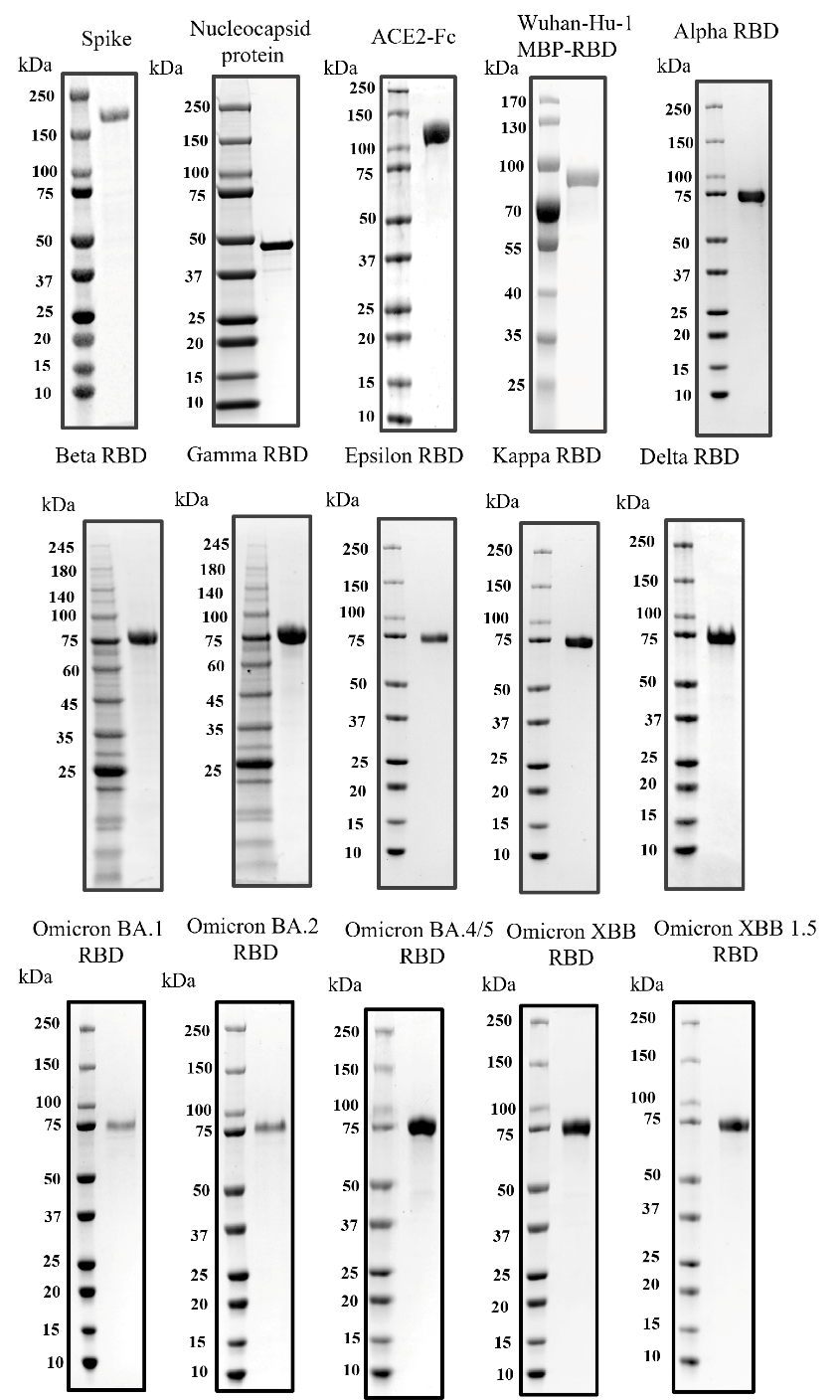


**Supplementary Fig. 1. Purified SARS-CoV-2 antigens and receptor protein.** Size of purified proteins were confirmed on SDS-PAGE gel. Proteins include Spike, RBD, and Nucleocapsid of SARS-CoV-2 Wuhan-Hu-1 strain, ACE2 receptor conjugated with IgG Fc region, and RBD of eleven SARS-CoV-2 variants.

**
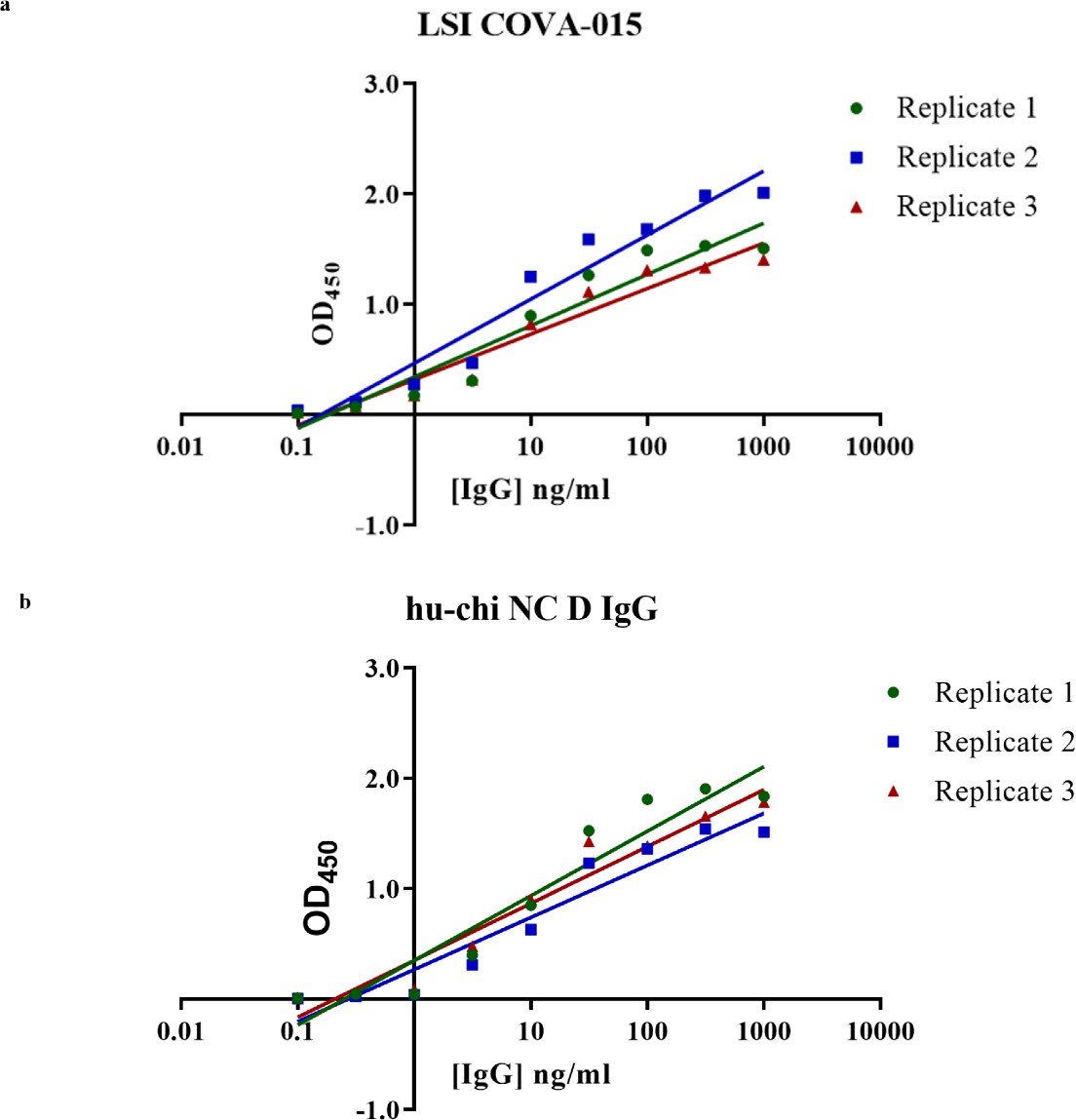
**

**Supplementary Figure. 2. IgG standard curves generated for Quantitative ELISA**

a. LSI-COVA-015 fully human monoclonal IgG was used for anti-Spike and anti-RBD IgG quantitation.

b. hu-chi NC D recombinant monoclonal human chimeric IgG was used for anti-Nucleocapsid IgG quantitation


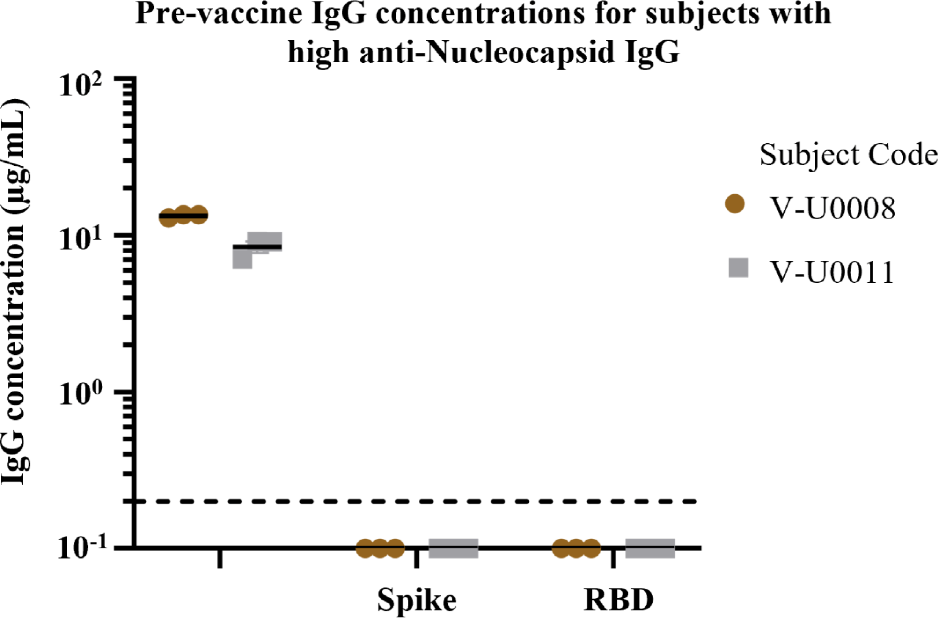


**Supplementary Figure. 3. Pre-vaccine IgG levels for subjects with high anti-Nucleocapsid IgG**

Pre-vaccination IgG levels against SARS-CoV-2 Spike and RBD were negative for two subjects with anti-high Nucleocapsid IgG concentrations.


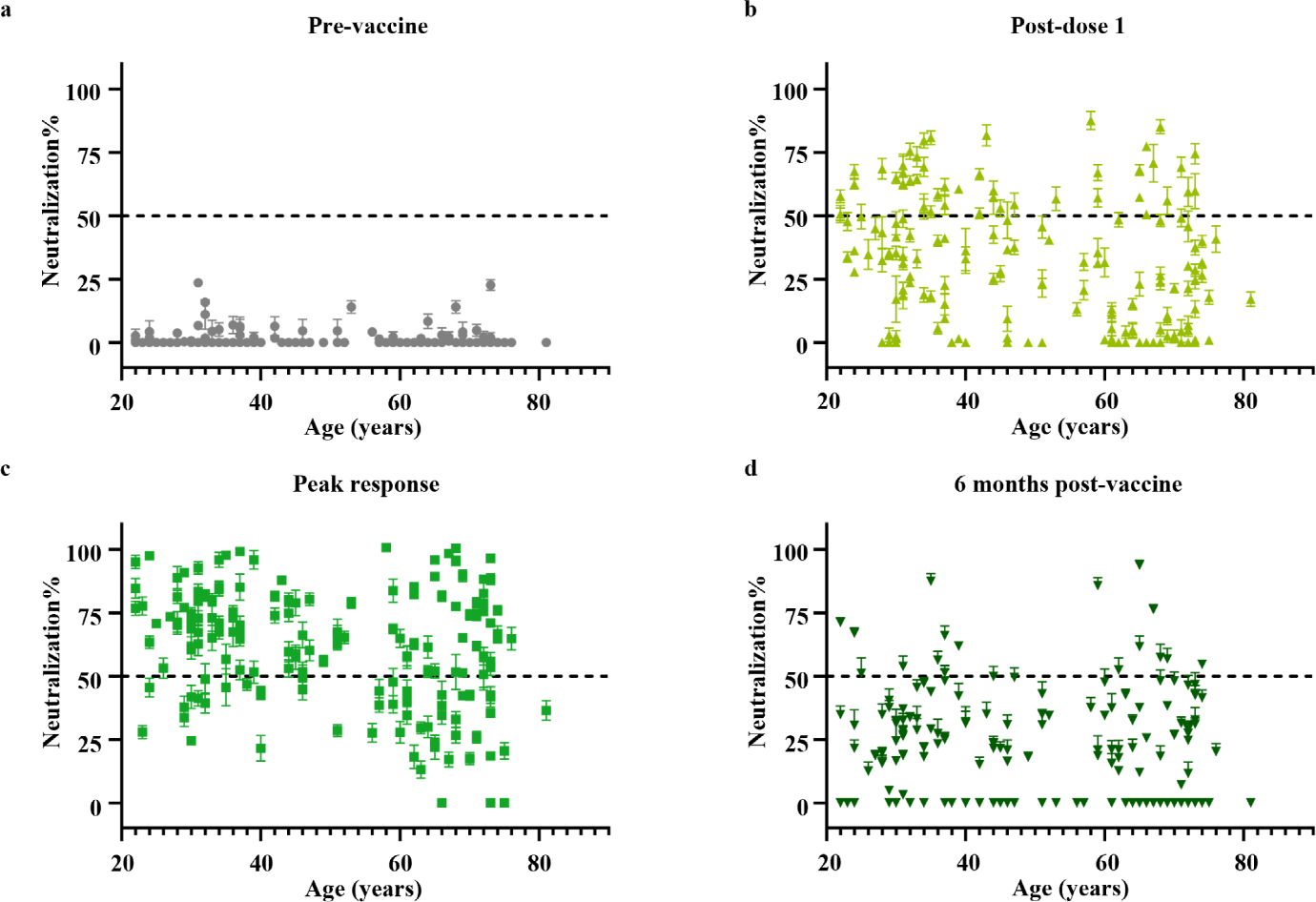


**Supplementary Figure. 4. Neutralizing response versus age.** Neutralizing response in all vaccinees were evaluated by PVNT and plotted against age (**a-d**) at four timepoints. N=168.


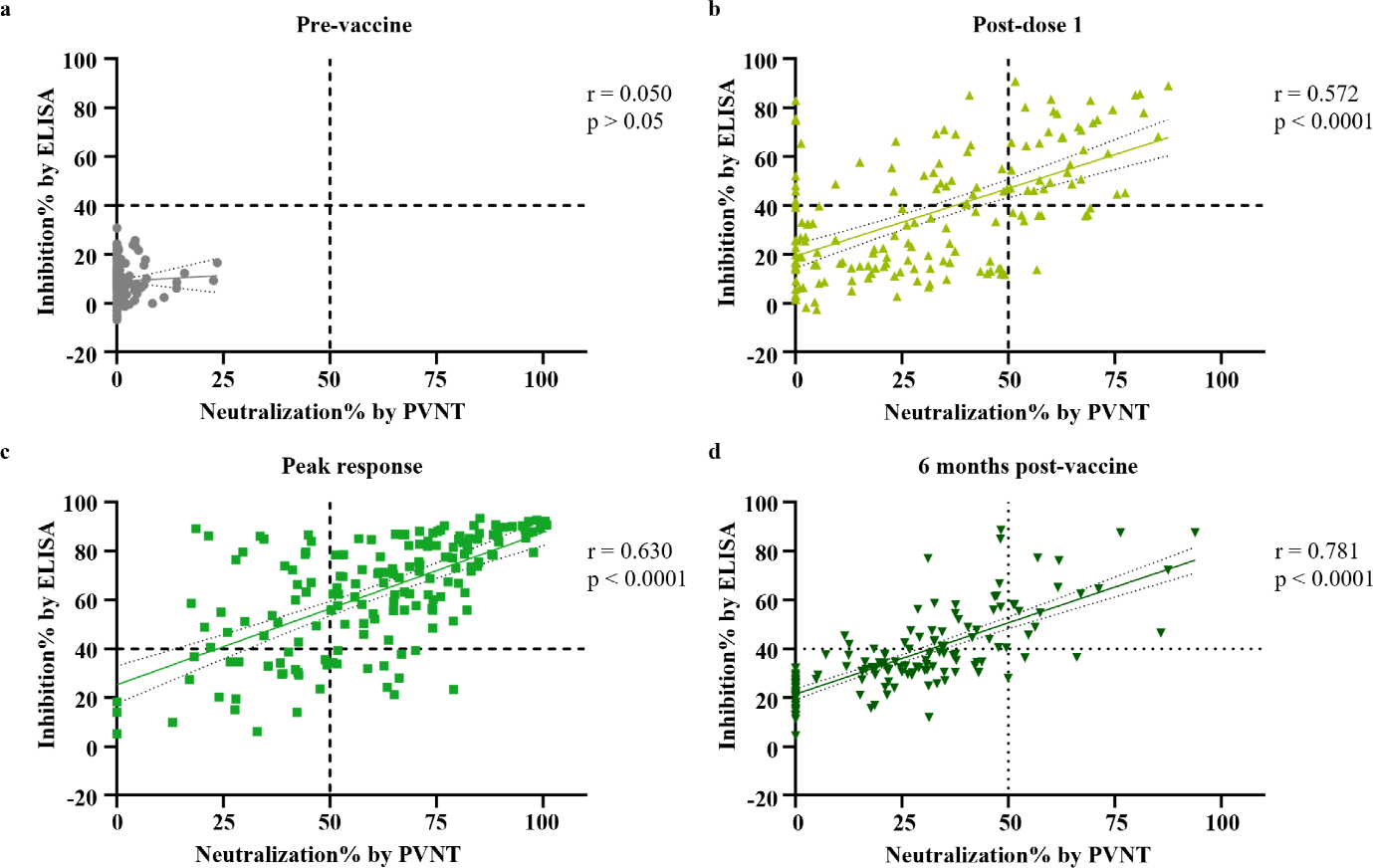


**Supplementary Figure. 5. Correlation between neutralizing response and ACE2 inhibitory response.** Association between neutralizing response by PVNT and ACE2-RBD binding inhibition for Wuhan-Hu-1 RBD (**a-d**) at four timepoints were modelled using simple linear regression. Pearson’s correlation coefficients and p-values are shown.


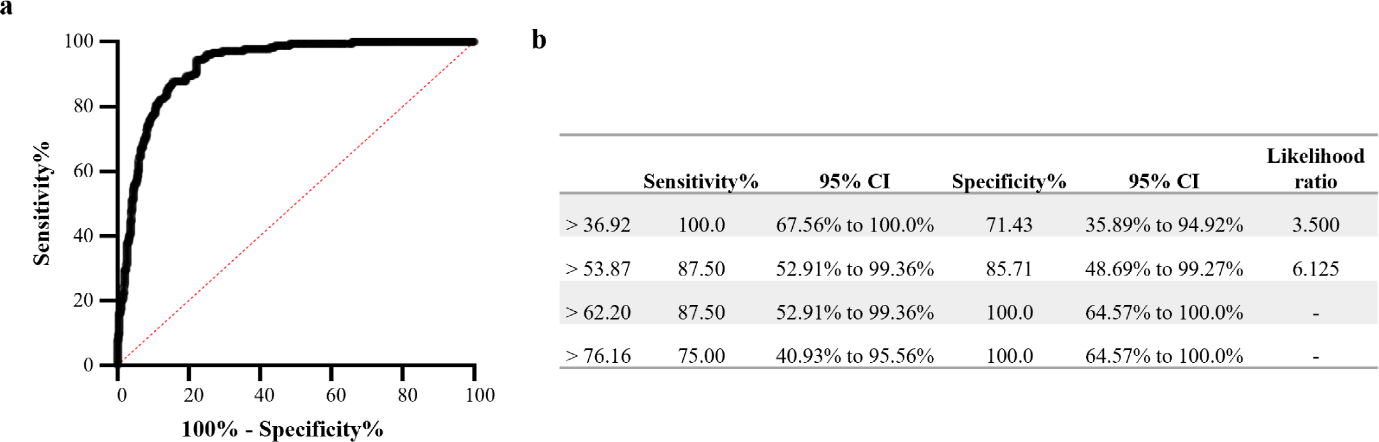


**Supplementary Fig. 6: Correlation between IC50, ACE2-RBD binding inhibition response, and PVNT. (a)** Using a threshold of 50% neutralization by PVNT, the predictability of ACE2-RBD binding inhibition response for defining neutralizers was evaluated with an ROC curve. N=168. **(b)** Sensitivity, specificity, and likelihood ratio of using ACE2 inhibition% as a predictor for an IC50 value of 150 IU/mL was evaluated. Figures close to the arbitrary threshold of 65% inhibition are reported.


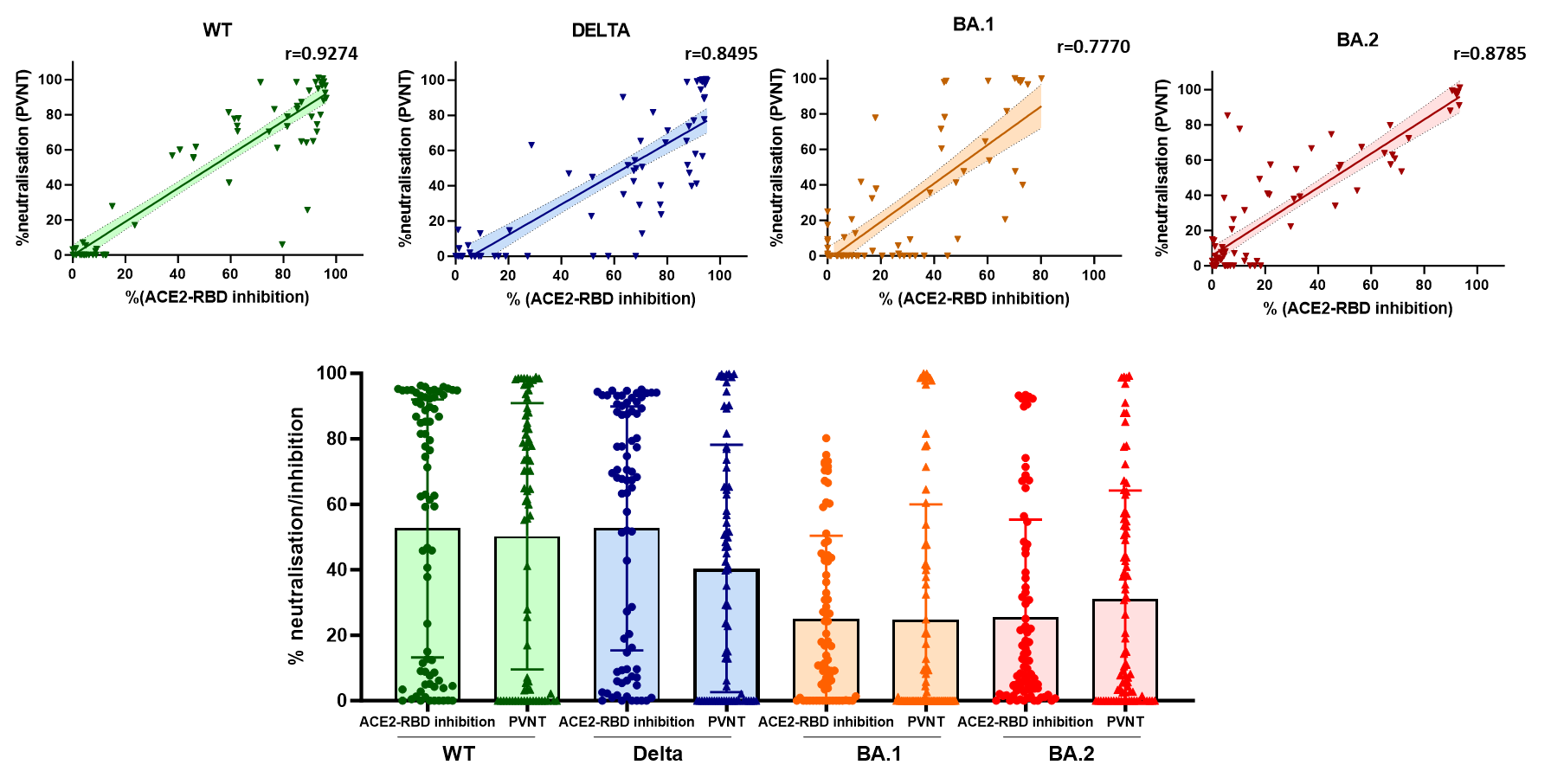


**(a)**

**(b)**

**(c)**

**(d)**

**(e)**

**Supplementary Fig. 7: Correlation between neutralising response and ACE2-RBD binding response.** Associations between neutralising responses by PVNT and ACE2-RBD binding inhibition for the major VOCs Wu-H1, Delta, BA.1 and BA.2 **(a-d)** were modelled using simple linear regression. Pearson’s correlation coefficients and p-values are shown. **(e)** The scatter plots with the bar graphs represent mean ± sd percentage neutralisation/inhibition values.

| K417N F | ATATTGCTGATTATAATTATAAATTACCAGATGA |
| --- | --- |
| K417 R | TTCCAGTTTGCCCTGGAGCGA |
| E484K F | AAAGGTTTTAATTGTTACTTTCCTTTA |
| E484K R | AACACCATTACAAGGTGTGCT |
| A570D F | ATGACACTACTGATGCTGTCCGT |
| A570D R | CAATGTCTCTGCCAAATTGTTGGA |
| N501Y F1 | GGTGTTGGTTACCAACCATACAGA |
| N501Y R1 | GTAAGTGGGTTGGAAACCATATGATTG |
| L452R F | AGGTATAGATTGTTTAGGAAGTCTAATCTC |
| L452R R | GTAATTATAATTACCACCAACCTTAGAA |
| K417TF | CTATTGCTGATTATAATTATAAATTACCAGATGA |
| E484K 417 R | TAAAGGAAAGTAACAATTAAAACCTTTAACACCATTACA |
| N501Y 417 F | CAATCATATGGTTTCCAACCCACTTACGGTGTTGGTTAC |
| E484Q F | CAAGGTTTTAATTGTTACTTTCCTTTA |
| T478K R | AACACCATTACAAGGTTTGCT |
| E484 F | GAAGGTTTTAATTGTTACTTTCCTTTA |
| D21 R | CAAAAGGGCACAAGTTTGTAATATTAGGAA |
| I14 F | ATTACAAACTTGTGCCCTTTTGATGAAG |
| G230 F | GCACAGGTGTTCTTACTGAGTCTAAC |
| E236 R | CTCAGTAAGAACACCTGTGCCTTTTAA |
| E236 T R | CTCAGTAAGAACACCTGTGCCTGTTAA |

**Supplementary Table 4. Primers.**

List of primers used to generate constructs for RBD variants.
