# Supplementary material for: Employment of a high throughput functional assay to define the critical factors that influence vaccine induced cross-variant neutralizing antibodies for SARS-CoV-2
Source: Sci Rep. 2023 Dec 9;13:21810. doi: 10.1038/s41598-023-49231-w (PMC10710454; doi:10.1038/s41598-023-49231-w)

**Supplementary Figure 1- Blots for gel images**

1. **RBDs**


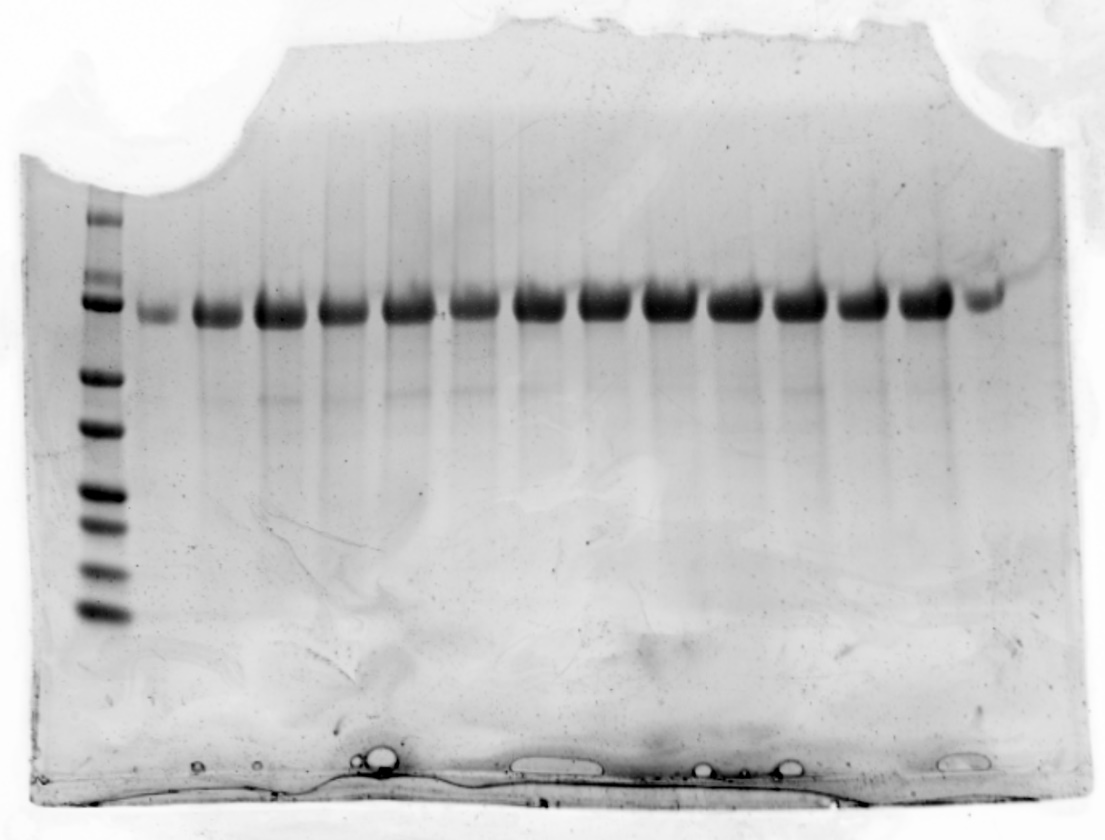


75 kda

**From left:**

Lane 1: Protein ladder

Lane 2: -

Lane 3: Wuhan-Hu-1 MBP RBD

Lane 4: Alpha RBD

Lane 5: Beta RBD

Lane 6: Gamma RBD

Lane 7: Delta RBD

Lane 8: Epsilon RBD

Lane 9: Kappa RBD

Lane 10: Omicron BA.1 RBD

Lane 11: Omicron BA.2 RBD

Lane 12: Omicron BA.4/5 RBD

Lane 13: XBB RBD

Lane 14: XBB 1.5 RBD

Lane 15: -

**ii) ACE2-Fc**

   
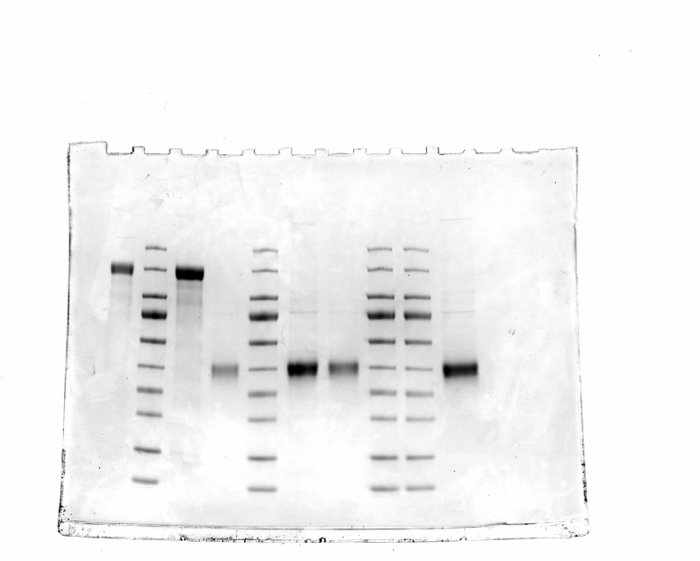


**iii) Hexapro spike**


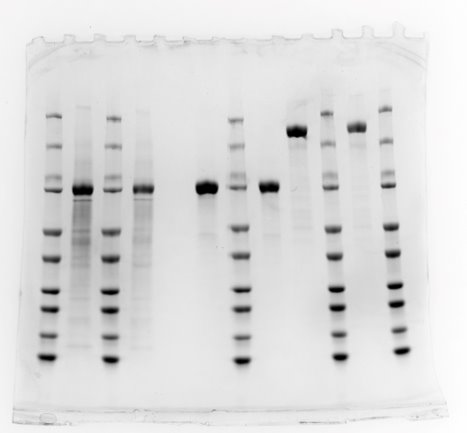


**iv) Nucleocapsid**


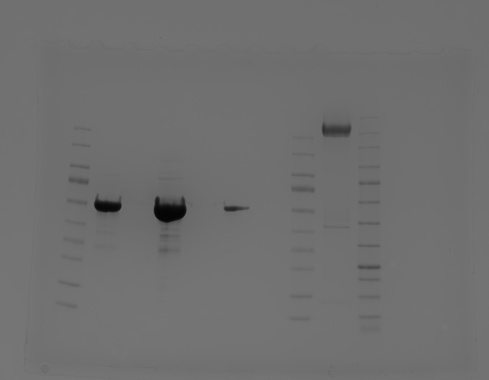

Supplement: Supplementary file 4 — Supplementary Information 4. [file 41598_2023_49231_MOESM4_ESM.docx]
