# Supplementary material for: Employment of a high throughput functional assay to define the critical factors that influence vaccine induced cross-variant neutralizing antibodies for SARS-CoV-2
Source: Sci Rep. 2023 Dec 9;13:21810. doi: 10.1038/s41598-023-49231-w (PMC10710454; doi:10.1038/s41598-023-49231-w)
Supplement: Supplementary file 5 — Supplementary Information 5. [file 41598_2023_49231_MOESM5_ESM.docx]

**Supplementary Figure 1- Original blots for gel images: revised file**

**Hexapro spike**


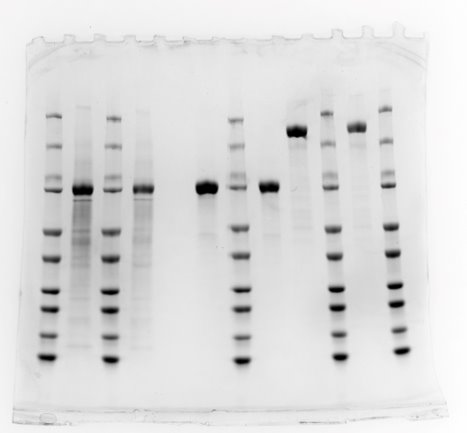


**Nucleocapsid**


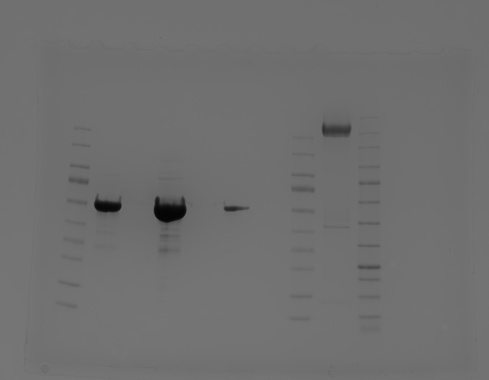


**ACE2-Fc**


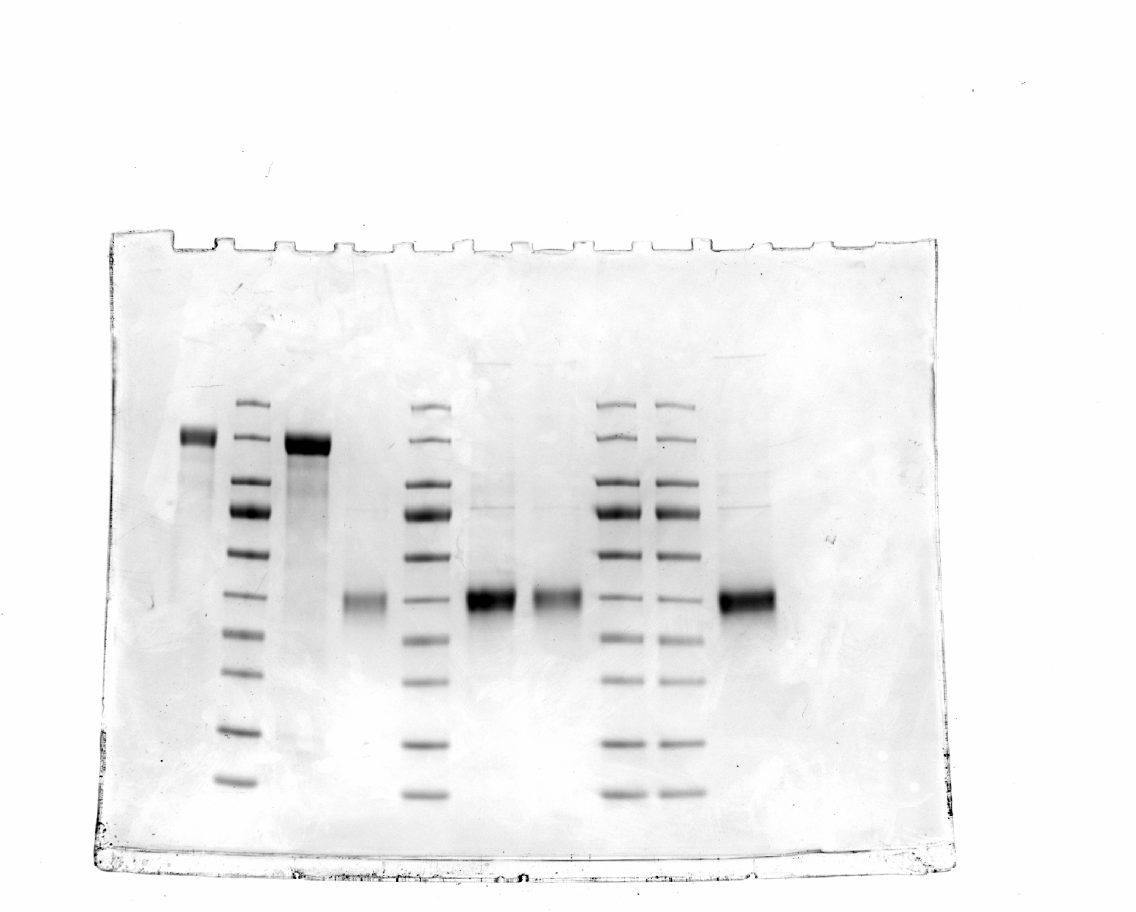


**Wuhan-Hu-1 MBP-RBD**


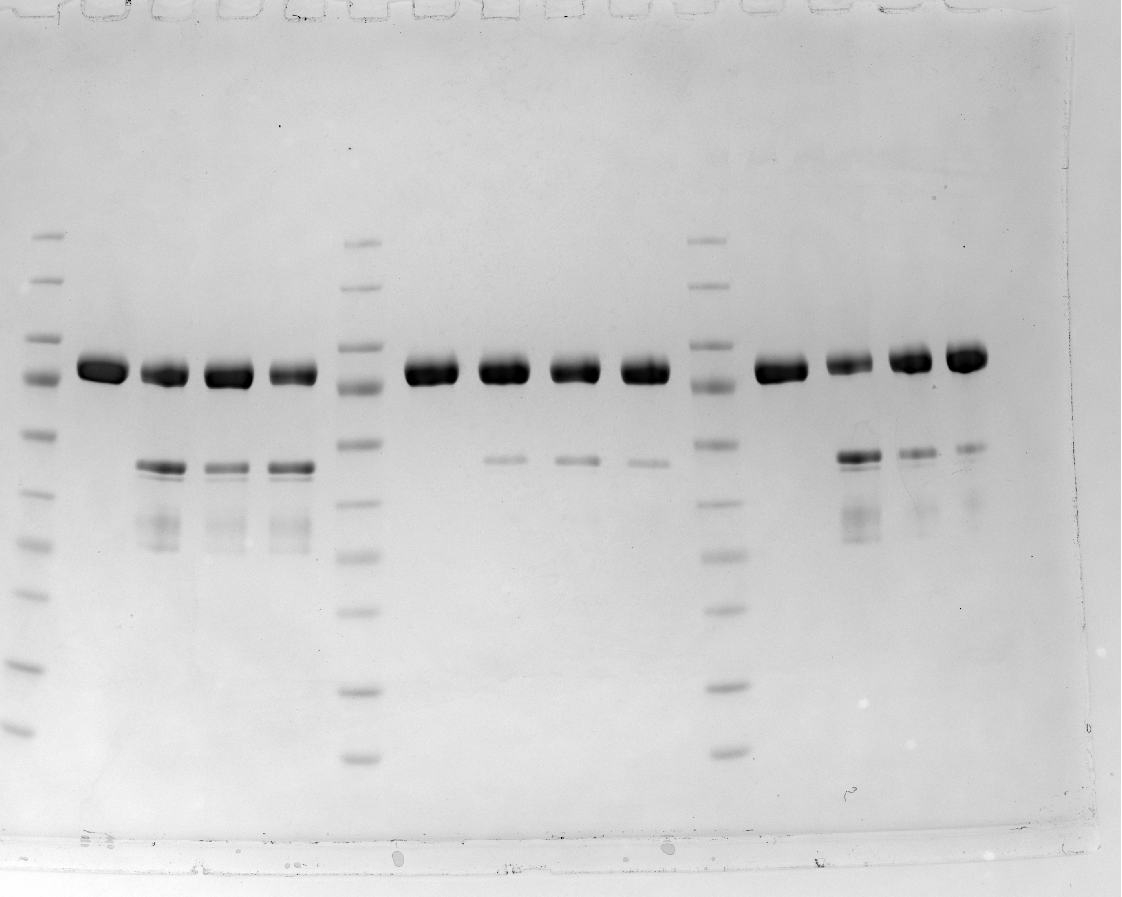


**Alpha RBD**


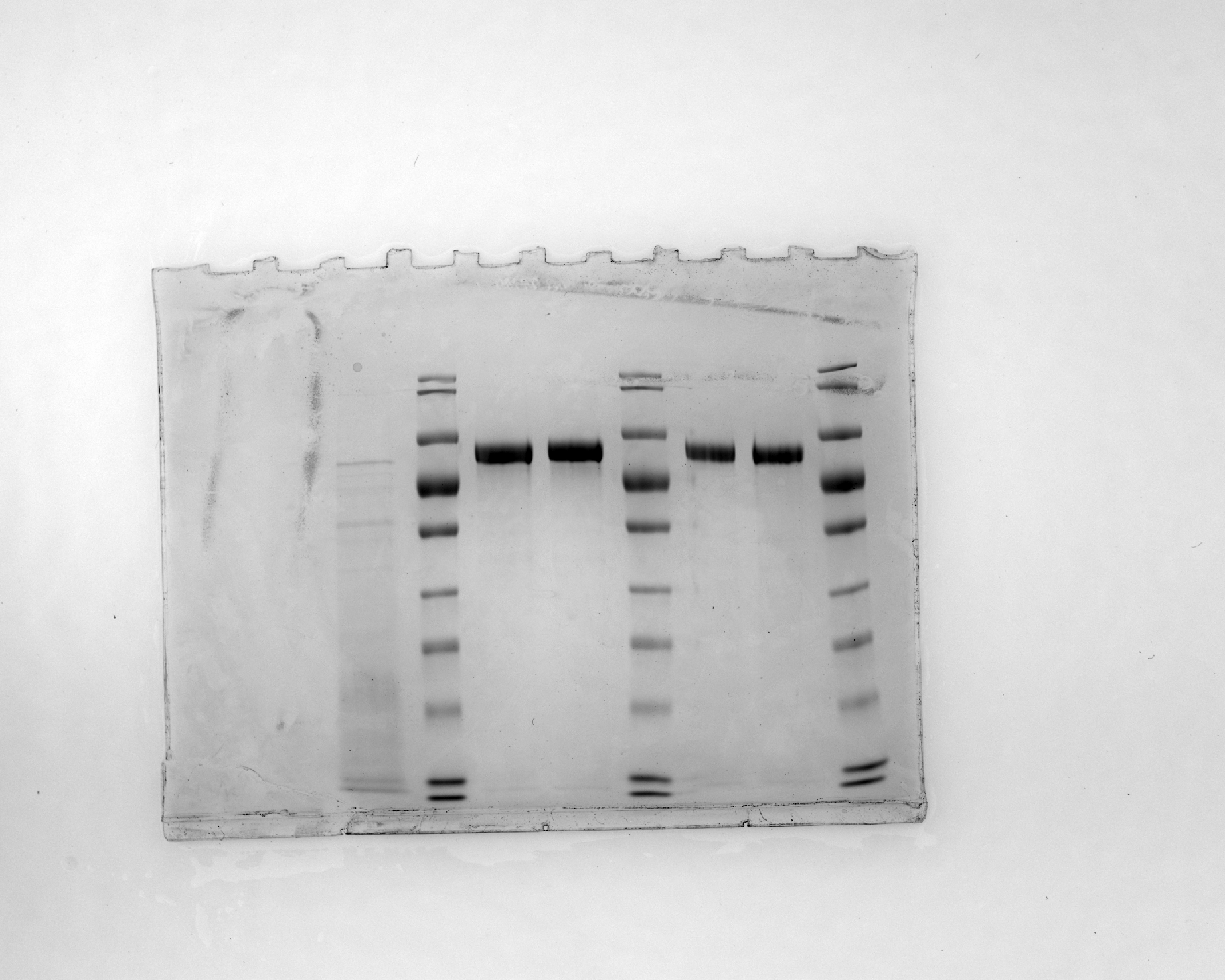


**Beta RBD**

**
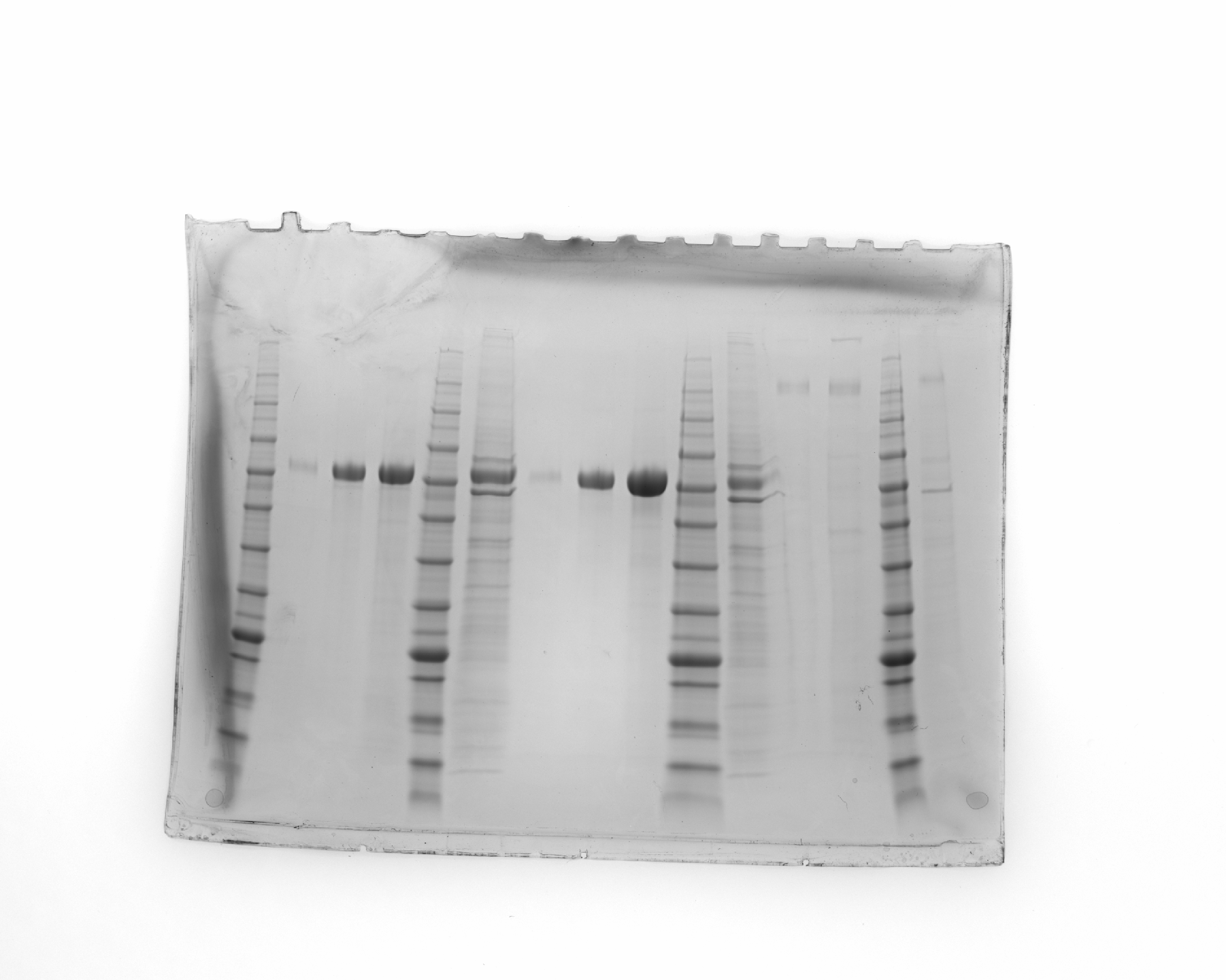
**

**Gamma RBD**

**
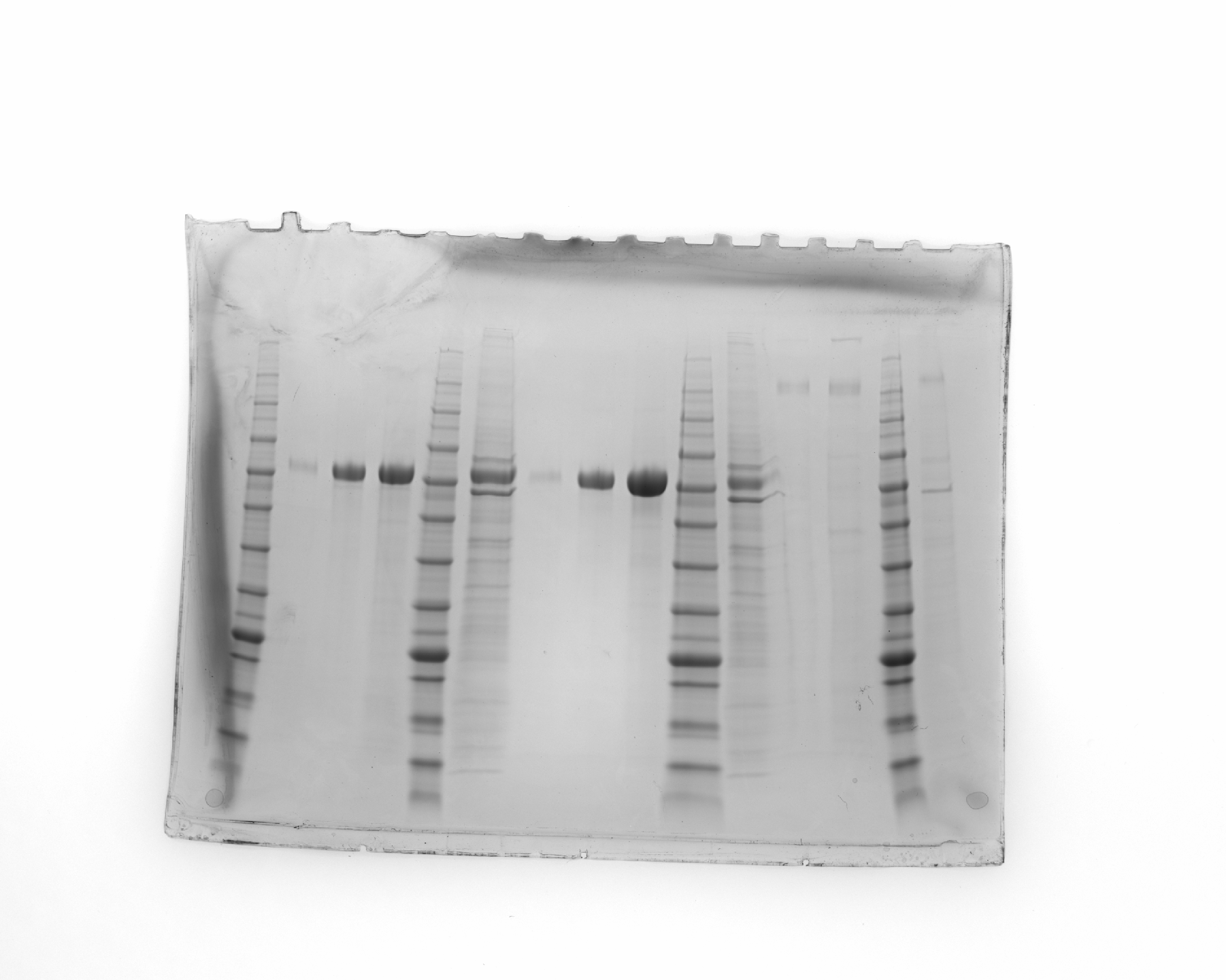
**

**Epsilon RBD**


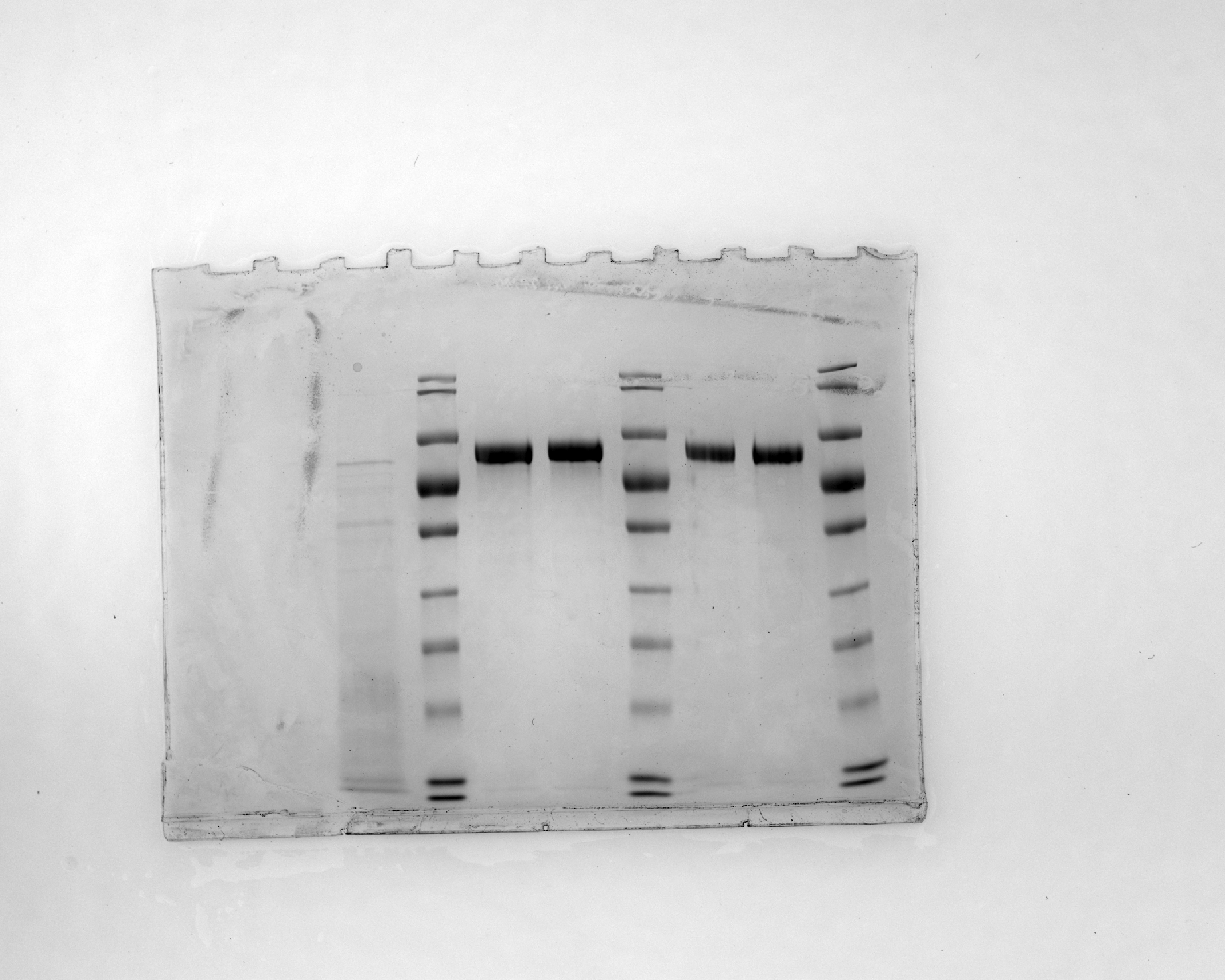


**Kappa RBD**

**
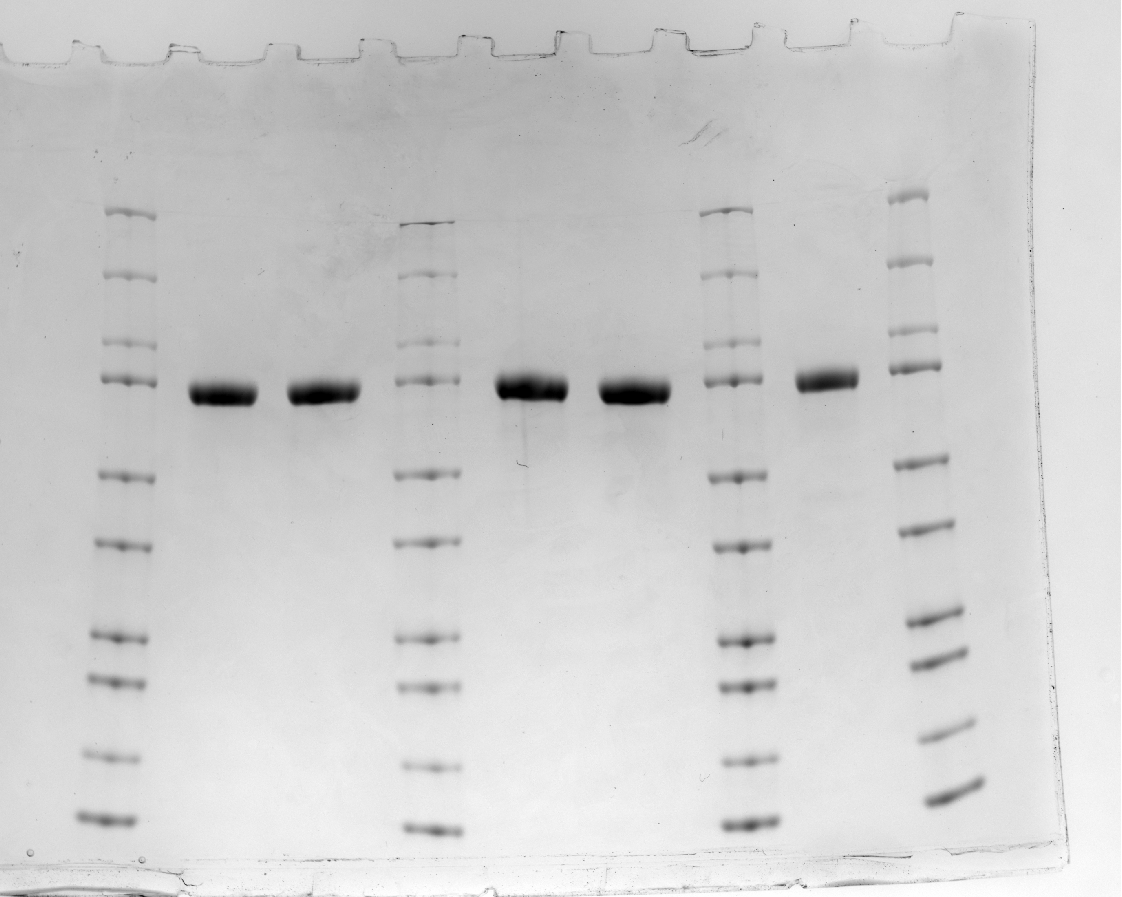
**

**Delta RBD**

**
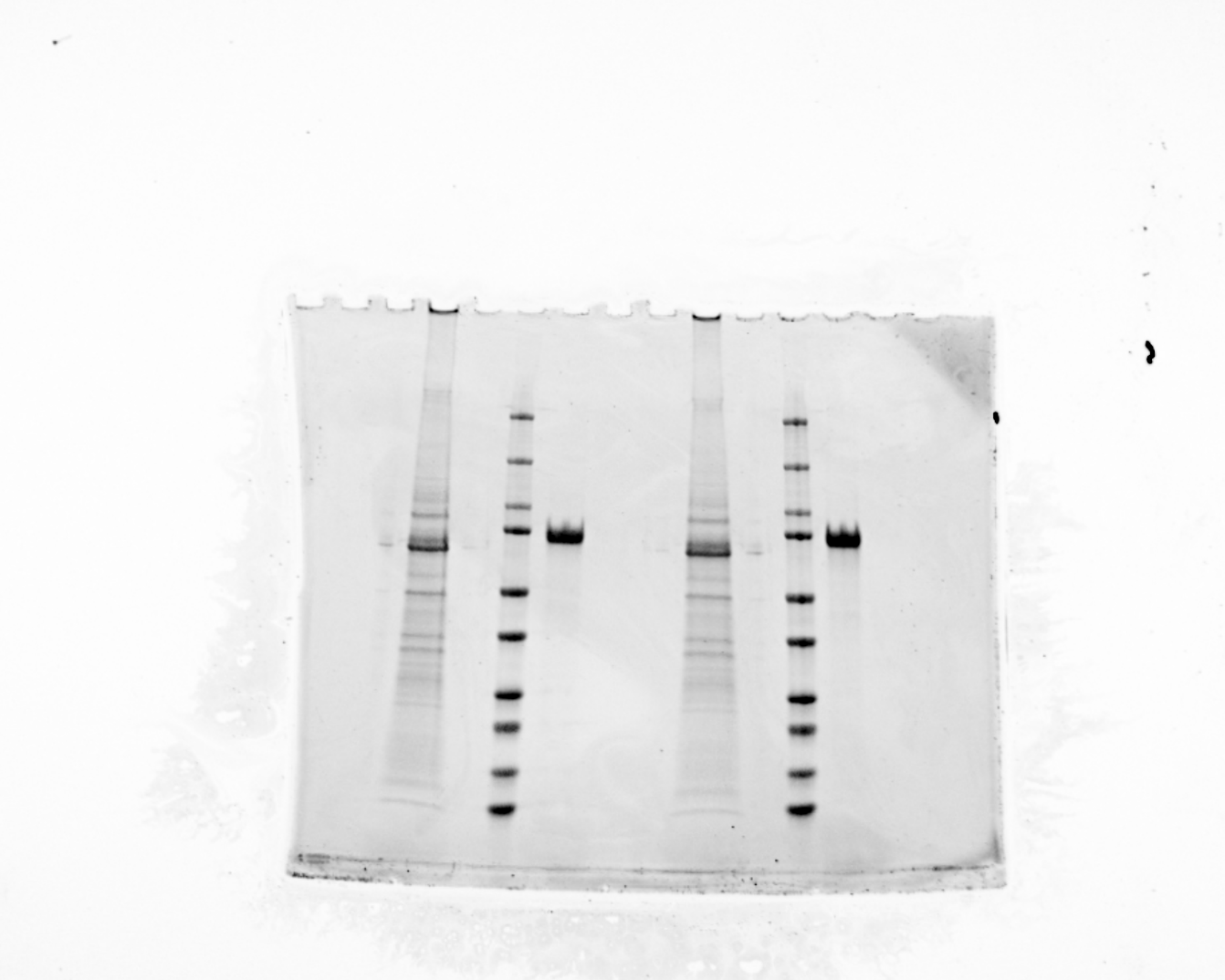
**

**Omicron BA.1**

**
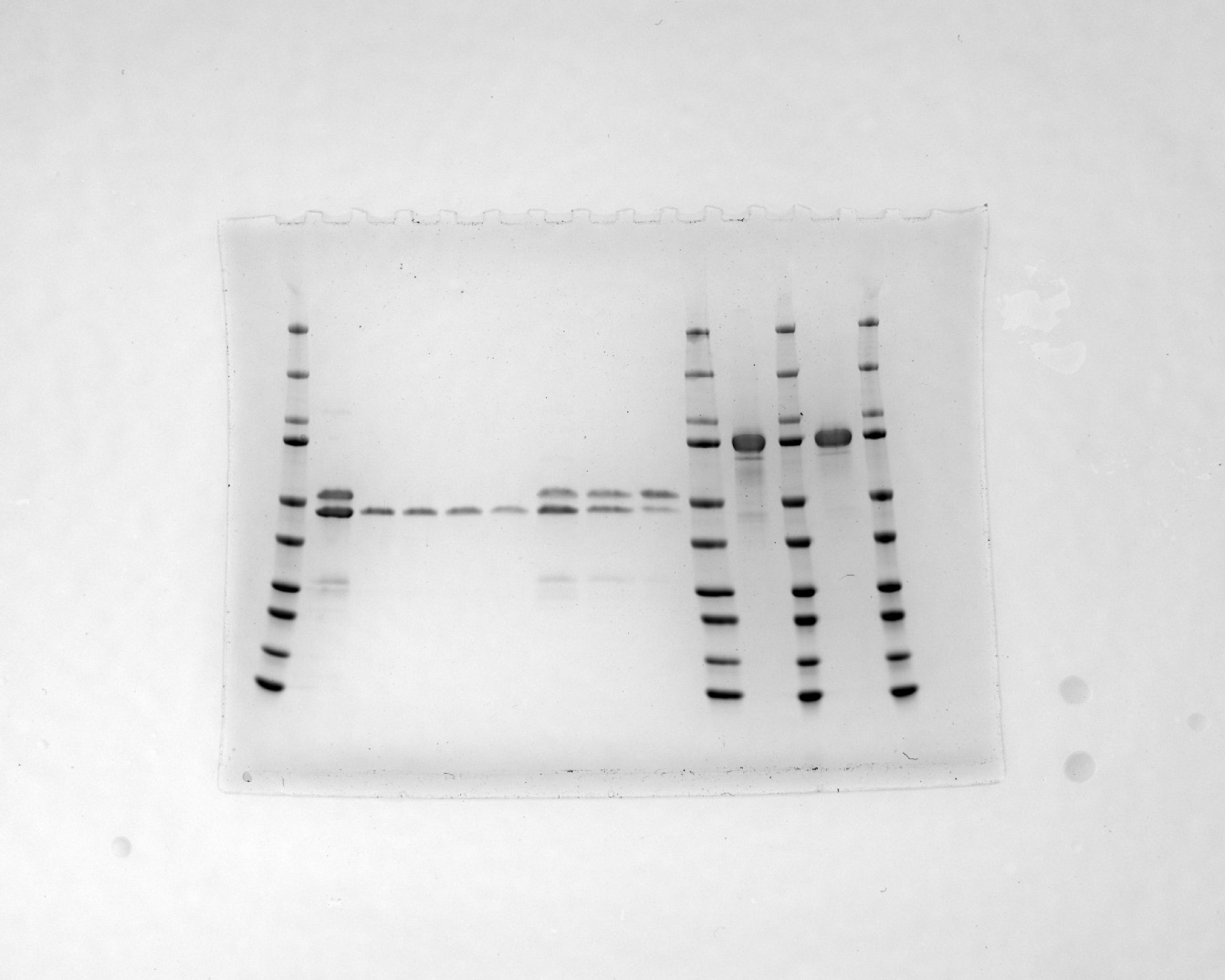
**

**Omicron BA.2**

**
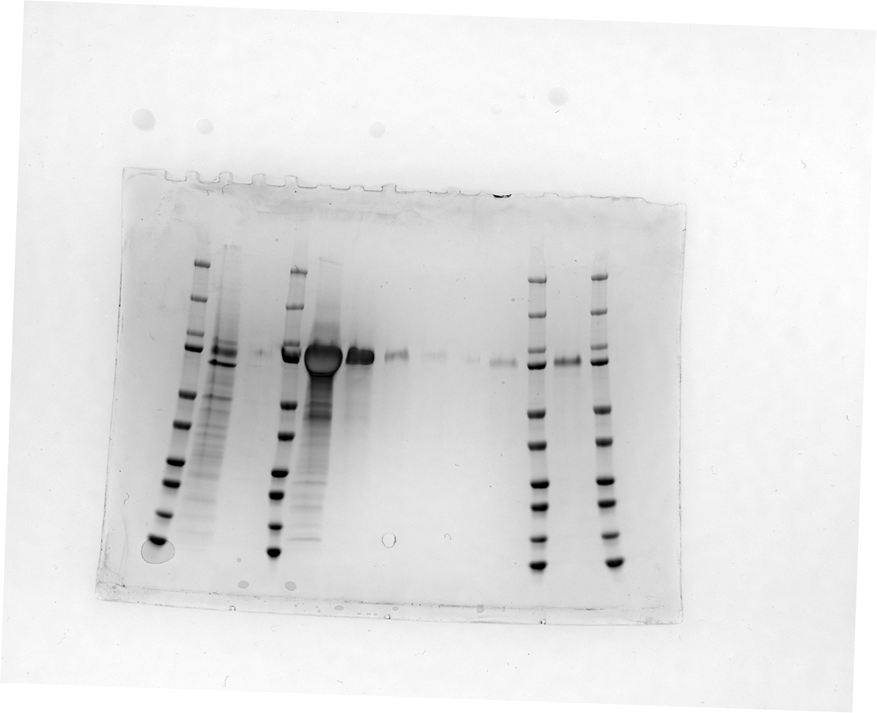
**

**Omicron BA.4/5**

**
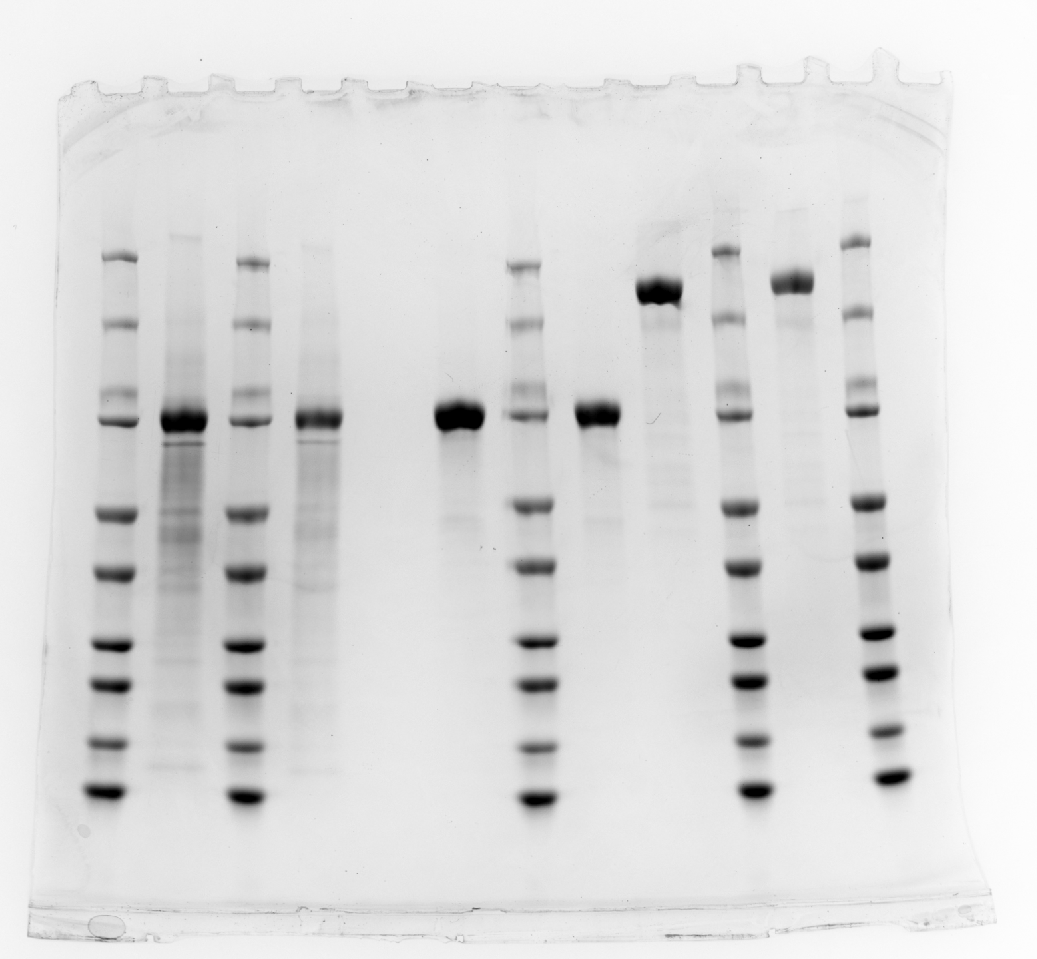
**

**Omicron XBB**

**
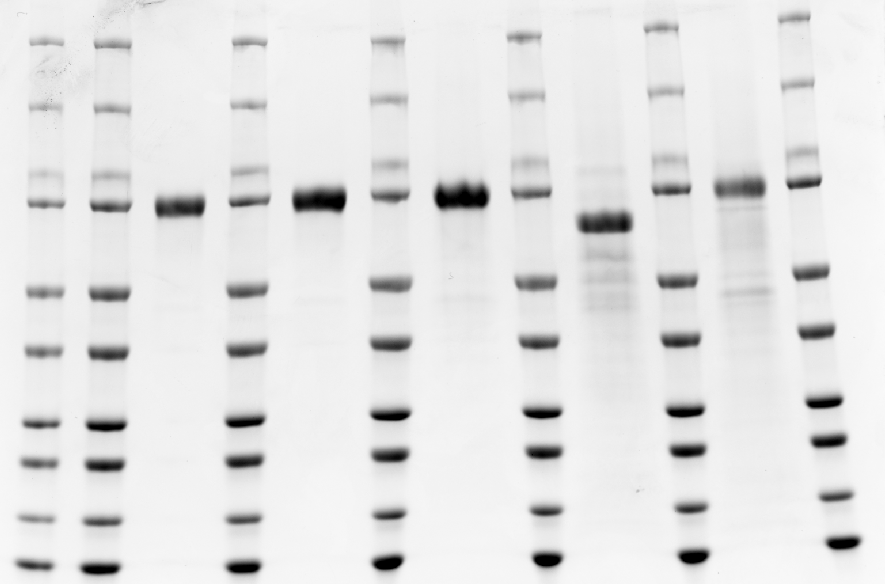
**

**Omicron XBB 1.5**

**
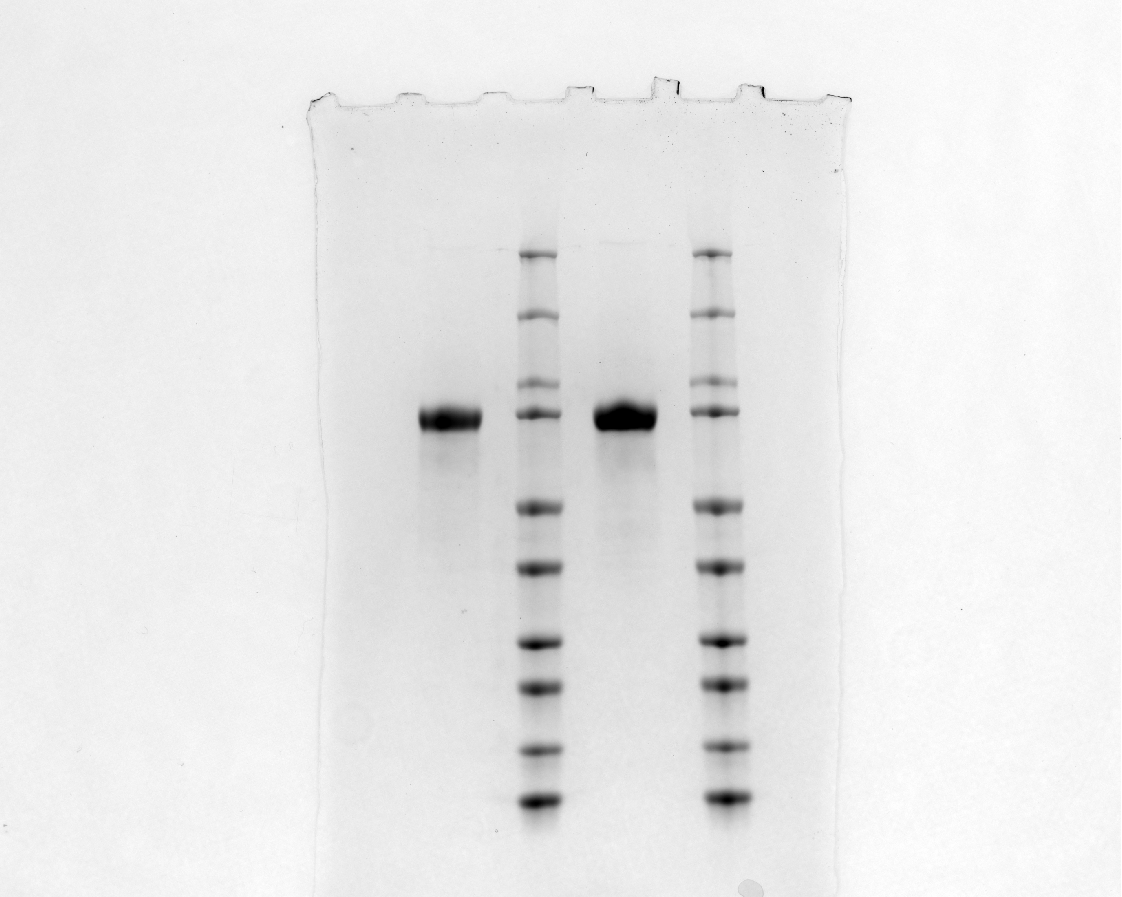
**
